# Enhanced Blood‐Brain Barrier Penetrability of BACE1 SiRNA‐Loaded Prussian Blue Nanocomplexes for Alzheimer's Disease Synergy Therapy

**DOI:** 10.1002/EXP.20230178

**Published:** 2025-03-07

**Authors:** Xiaoyuan Ding, Yanyu Hu, Xiaotong Feng, Zekun Wang, Qile Song, Chunxue Dai, Bangjia Yang, Xiaoyan Fu, Dongdong Sun, Cundong Fan

**Affiliations:** ^1^ School of Life Sciences Anhui Agricultural University Hefei China; ^2^ College of Biotechnology and Pharmaceutical Engineering West Anhui University Luan China; ^3^ Shandong Provincial Key Medical and Health Lab of Brain Injury and Functional Rehabilitation The Second Affiliated Hospital of Shandong First Medical University Taian Shandong Province China

**Keywords:** Alzheimer's disease, amyloid‐β, BACE1, blood‐brain barrier, Prussian blue nanoparticles

## Abstract

Amyloid‐β (Aβ) deposition was an important pathomechanisms of Alzheimer's disease (AD). Aβ generation was highly regulated by beta‐site amyloid precursor protein cleaving enzyme 1 (BACE1), which is a prime drug target for AD therapy. The silence of BACE1 function to slow down Aβ production was accepted as an effective strategy for combating AD. Herein, BACE1 interfering RNA, metallothionein (MT) and ruthenium complexes ([Ru(bpy)_2_dppz]^2+^) were all loaded in Prussian blue nanoparticles (PRM‐siRNA). PRM‐siRNA under near‐infrared light irradiation showed good photothermal effect and triggered instantaneous opening of blood‐brain barrier (BBB) for enhanced drug delivery. BACE1 siRNA slowed down Aβ production and Cu^2+^ chelation by metallothionein (MT) synergistically inhibited Aβ aggregation. Ruthenium (Ru) could real‐timely track Aβ degradation and aggregation. The results indicated that PRM‐siRNA significantly blocked Aβ aggregation and attenuated Aβ‐induced neurotoxicity and apoptosis in vitro by inhibiting ROS‐mediated oxidative damage and mitochondrial dysfunction through regulating the Bcl‐2 family. PRM‐siRNA in vivo effectively improved APP/PS1 mice learning and memory by alleviating neural loss, neurofibrillary tangles and activation of astrocytes and microglial cells in APP/PS1 mice by inhibiting BACE1, oxidative damage and tau phosphorylation. Taken together, our findings validated that BACE1 siRNA‐loaded Prussian blue nanocomplexes showed enhanced BBB penetrability and AD synergy therapy.

## Graphical abstract

BACE1 interfering RNA, metallothionein and ruthenium complexes ([Ru(bpy)2dppz]^2+^) were all loaded in prussian blue nanoparticles (PRM‐siRNA). PRM‐siRNA under near‐infrared light irradiation showed good photothermal effect and triggered instantaneous opening of blood‐brain barrier for enhanced drug delivery. BACE1 siRNA slowed down Aβ production, and Cu^2+^ chelation by metallothionein synergistically inhibited Aβ aggregation, and finally improved APP/PS1 mice learning and memory.

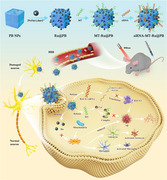

## 1 Introduction

Alzheimer's disease (AD), commonly known as dementia, is a progressive neurodegenerative disease that usually results in memory loss, poor judgment, mood swings and aphasia in AD patients. At present, Alzheimer's disease patients account for more than half of the dementia population and show a growing trend of youthfulness. But the specific pathological mechanism leading to AD has not been fully explained. AD patients have a large accumulation of β‐amyloid (Aβ) in the brain, and this accumulation of Aβ in the brain produces reactive oxygen species (ROS) that trigger oxidative damage to cellular components, such as DNA, lipids and proteins. Meanwhile, Cu^2+^, Zn^2+^, and Fe^2+^ are abundantly enriched in the area around senile plaques [1, 2, 3, 4]. Current research generally indicates that metal ions can induce Aβ aggregation and play an important regulatory role in the process of Aβ aggregation into fibers [5, 6, 7]. The aggregation of metal ions and Aβ enhances the neurotoxicity of Aβ [8, 9, 10, 11]. Additionally, the presence of the blood brain barrier (BBB) is a major obstacle to the treatment of AD. While, the BBB effectively blocks harmful substances from entering the brain, it also blocks over 99% of conventional drugs, resulting in poor efficacy of drugs used to treat neurodegenerative diseases. But currently, clinical treatment for AD has not shown significant therapeutic effects in slowing down disease progression. Therefore, the development of novel AD therapeutics is imperative.

Aggregated amyloid peptide (Aβ), hyperphosphorylation of tau protein, and neuroinflammation are the key pathological features of AD. The amyloid protein (APP) is affected by the BACE‐1 gene, and the abnormal accumulation of Aβ is caused by the sequential cleavage of amyloid precursor protein (APP). Impaired BACE‐1 (β‐site APP cleavage enzyme 1) and γ secretase activity is considered a key causative event in AD [12, 13, 14, 15]. Therefore, the strategy of reducing the activity of BACE‐1 and thereby reducing the level of Aβ is considered a potential treatment for AD [16, 17]. By blocking the expression of disease‐causing genes, small interfering RNA (siRNA) has high target specificity, a low effective dose and a relatively simple drug development process, providing a promising treatment method for the treatment of brain diseases [18, 19, 20, 21]. It has been reported that the delivery of small interfering RNA (siRNA‐BACE‐1) to the brains of mice through tail vein injection can partially reduce the neuropathological features of AD [22, 23, 24]. Besides, recent studies have shown that nanodelivery methods have great potential to overcome these challenges [19].

Nanoparticles have unique structural advantages, easy surface functionalization, easy modification and the ability to cross the BBB and thus have attracted wide attention for the treatment of AD. Prussian blue (PB) is a clinical drug approved by the FDA for the treatment of thallium poisoning or radiation exposure [25, 26]. Due to the unique advantages of Prussian blue, such as similarity in size with biomolecules, high surface‐to‐volume ratio, easy surface modification and functionalization, excellent solubility, and stability, it has promising applications in biomedical fields [27, 28, 29, 30]. Prussian blue‐like nanoparticles have good light‐to‐heat conversion efficiency under near‐infrared irradiation (NIR) and may have certain advantages in improving BBB permeability [31, 32, 33, 34, 35]. Besides, a large number of experiments have demonstrated that under the irradiation of NIR light, photothermal nanomaterials can convert laser energy into thermal energy, leading to localized high temperatures, which can disrupt the structure of Aβ fibrils and interfere with their growth, thus making PB a nanomaterial with great potential for the treatment of AD [36, 37, 38, 39]. Metallothionein (MT) is currently used to chelate metal ions and has been reported in many studies [40, 41]. MT can chelate Cu^2+^ to inhibit nerve cell damage and enhance neuromodulation. MT has great biological significance in a variety of neurodegenerative brain diseases (such as AD) [42, 43]. The combination of [Ru(bpy)_2_dppz]^2+^ with Aβ fibers will produce specific fluorescence [44], which can be used to real‐timely track the Aβ degradation and aggregation.

**SCHEME 1:**
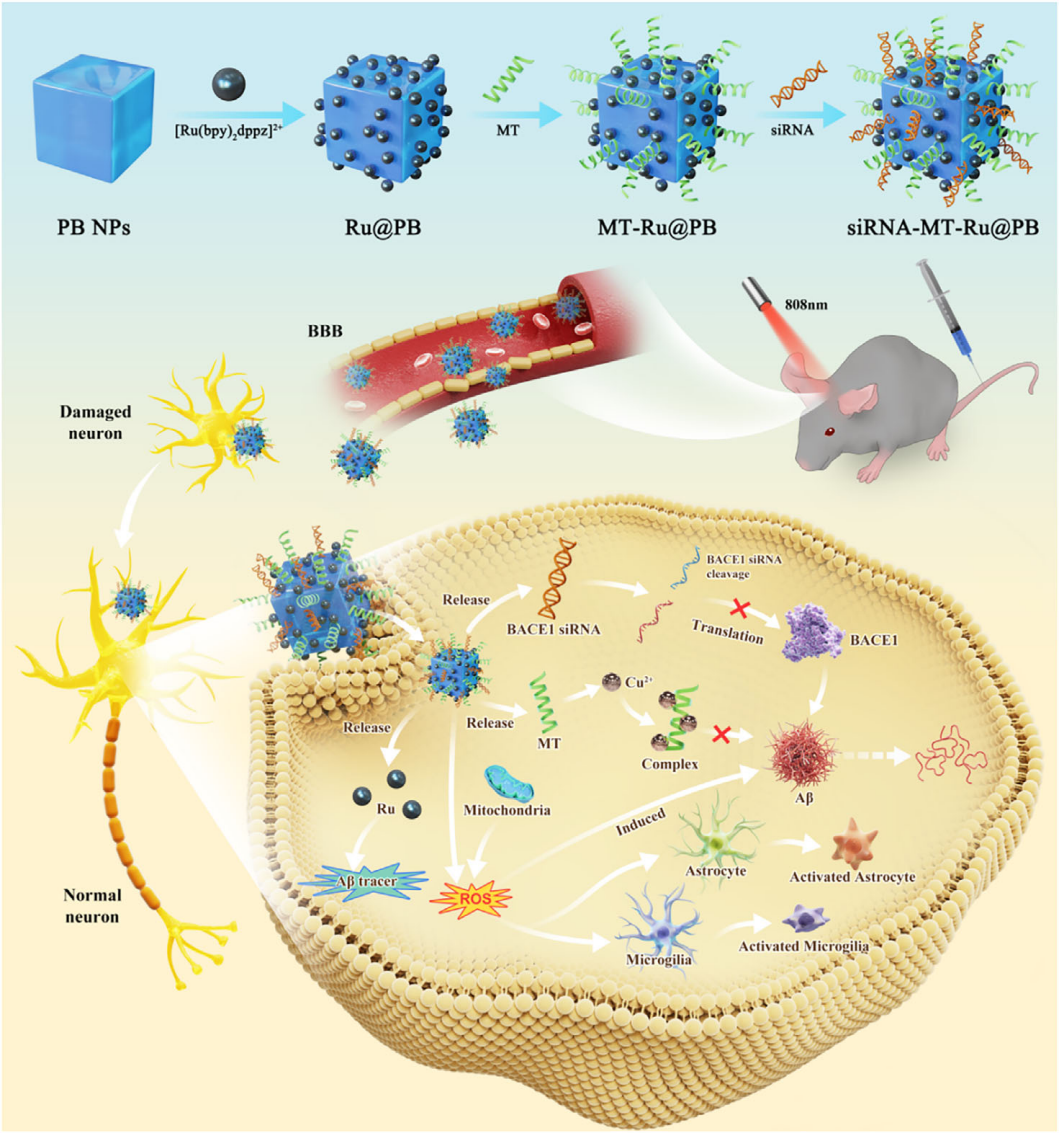
Synthesis route and proposed signal mechanism of PRM‐siRNA NPs against AD.

In the present study, small interfering RNA (BACE1 siRNA), metallothionein (MT) and ruthenium complexes ([Ru(bpy)_2_dppz]^2+^) were all loaded in Prussian blue nanoparticles (PRM‐siRNA). PRM‐siRNA under NIR triggered instantaneous BBB opening for enhanced drug delivery. BACE1 siRNA slowed down Aβ production, and MT chelated Cu^2+^, which synergistically inhibited Aβ aggregation. [Ru(bpy)_2_dppz]^2+^ real‐timely tracked Aβ degradation and aggregation. The results indicated that PRM‐siRNA treatment significantly blocked Aβ aggregation and subsequently attenuated Aβ‐induced neurotoxicity and apoptosis in PC12 and primary neurons by inhibiting ROS‐mediated oxidative damage and mitochondrial dysfunction through regulating Bcl‐2 family expression. PRM‐siRNA administration in vivo effectively improved APP/PS1 mice learning and memory by alleviating neural loss, neurofibrillary tangles, and activation of astrocytes and microglial cells in APP/PS1 mice by inhibiting BACE1 expression, oxidative damage, and tau phosphorylation. Taken together, our findings validated the rational design that BACE1 siRNA‐loaded Prussian blue nanocomplexes displayed enhanced BBB penetrability and synergy therapy for human AD. (Scheme 1)

## 2 Results

### 2.1 Synthesis and Characterization of PRM‐siRNA

**FIGURE 1:**
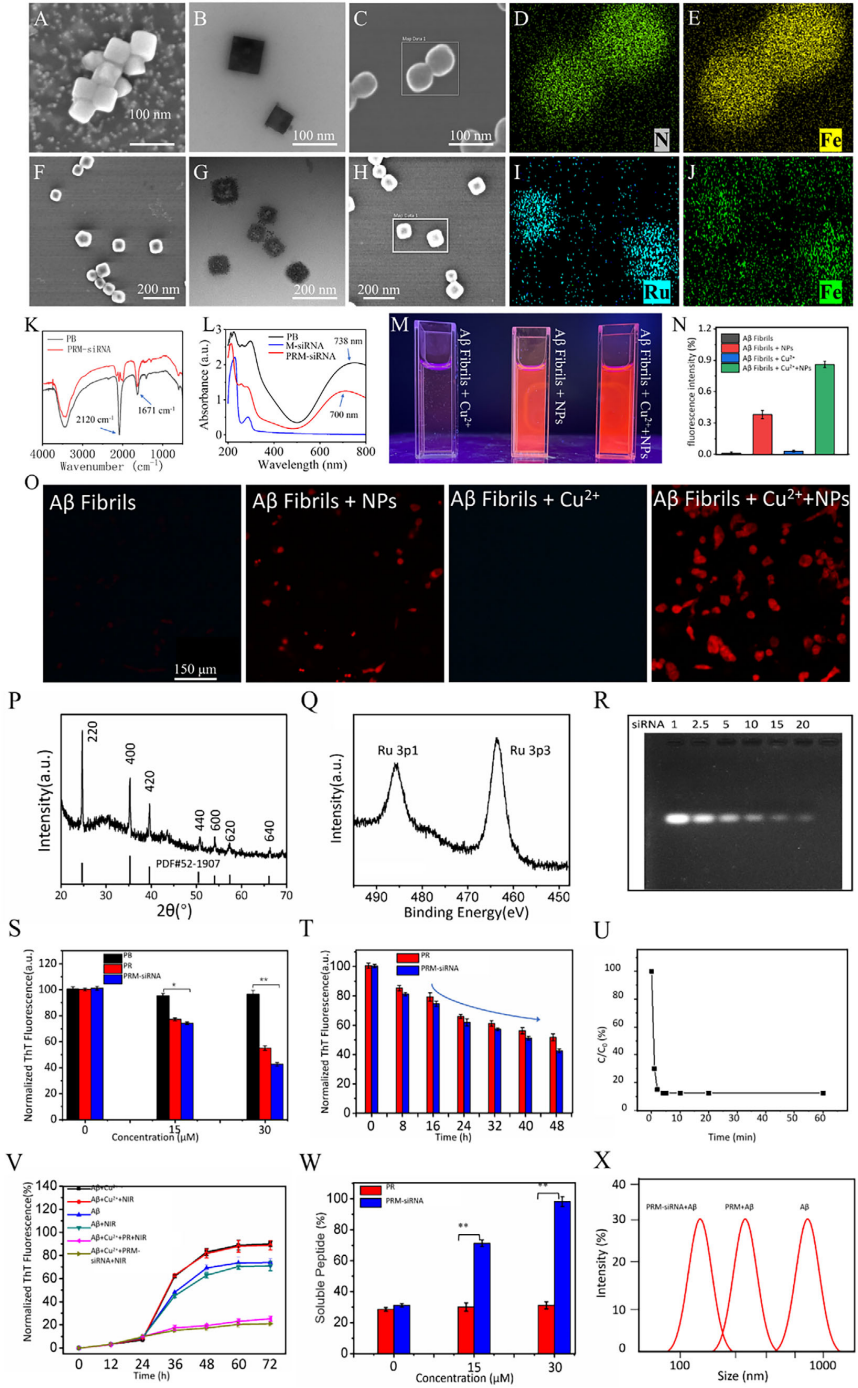
Characterization of PB NPs and PRM‐siRNA. Physicochemical properties of PRM‐siRNA. (A,B) SEM images and TEM images of (C) PB NPs respectively. (D,E) EDX mapping analysis of PB NPs. (F,G) SEM images and TEM images of (H) PRM‐siRNA NPs, respectively. (I,J) Mapping analysis of PRM‐siRNA NPs. (K) FT‐IR spectra of PB NPs and PRM‐siRNA NPs. (L) UV–vis absorption spectra of PB NPs, M‐siRNA (MT‐siRNA) and PRM‐siRNA NPs. (M) Fluorescence changes of Aβ fibrils and different NPs binding solutions. (N) Relative fluorescence intensity changes. (O) Fluorescence changes of Aβ fibrils combined with different NPs in PC12 cells. Each value represents the mean standard deviation (*n* = 3). (P) XRD analysis of PRM‐siRNA NPs. (Q) XPS analysis of PRM‐siRNA NPs. (R) Agarose gel retardation assay of PRM‐siRNA NPs. (S) Changes in normalized ThT fluorescence of preformed Aβ aggregates were incubated with different concentrations of nanoparticles for 48 h at 30°C. (T) The normalized ThT fluorescence of Aβ aggregates in the presence of PR (30 µM) and PRM‐siRNA NPs (30 µM) under different incubation durations. (U) Change of free Cu ion concentration in solution within 1 h after adding PRM‐siRNA NPs. *C*_0_ is the initial concentration of Cu ion, and *C* is the concentration of Cu ion at different time points. (V) Fibrillation kinetics of Aβ_42_ monomer by the development of ThT binding in the presence of PR, PRM, and PRM‐siRNA NPs under NIR or without NIR illumination. (W) The percentage of soluble Aβ_42_ monomer with different nanoparticles and different concentrations of nanoparticles incubated at 30°C for 48 h. (X) DLS measures the change in particle size after treatment with different drugs. The average particle size of Aβ aggregates is about 900–1000 nm, and the particle size after drug treatment is about 150 nm, which is similar to the particle size of the drug itself.

From Figure 1A,B, it can be seen that the synthesized Prussian blue nanoparticles are uniformly shaped cubes with a size of approximately 60 nm. EDX spectroscopy analysis shows the elemental distribution of Prussian blue nanoparticles (Figure 1C–E). Figure 1F,G shows that PRM‐siRNA is covered by nanoparticles, presumably formed by the modification of ruthenium complexes on the surface of PB nanoparticles. The EDX spectra (Figure 1H–J) show the elemental distribution of PRM‐siRNA NPs. The FTIR spectrum (Figure 1K) at 1671 cm^−1^ is the absorption peak of the amide stretching vibration in MT, while 2120 cm^−1^ is the absorption peak of the stretching vibration peak of the carbon and nitrogen triple bond. All these experimental results indicated that siRNA and MT were successfully loaded into the PR. The UV spectrum is shown in Figure 1L. PB has a characteristic UV peak of Prussian blue nanoparticles at 700 nm, and PRM‐siRNA has a significant blueshift compared with the UV spectrum of PB, which may be caused by the addition of siRNA and MT. Changes in Zeta potential and hydrodynamic size analysis also illustrate the successful construction of PRM‐siRNA (Figure S1).

[Ru(bpy)_2_dppz]^2+^ on PRM‐siRNA NPs can specifically bind to the aggregation products of β amyloid (Aβ) and produce fluorescence [44]. In both solution and cells, PRM‐siRNA fluoresce when bound to Aβ fibril, whereas Aβ fibril alone does not fluoresce (Figure 1M,O). As shown in Figure 1N, the fluorescence of the experimental group of Aβ+Cu^2+^+NPs was stronger than that of the experimental group of Aβ+NPs, which may be due to the addition of Cu^2+^ enhancing the aggregation of Aβ fibrils. The crystal structure of PRM‐siRNA was determined by X‐ray diffraction, and the XRD pattern showed that the position of the diffraction peak of PRM‐siRNA was consistent with the Prussian blue nanoparticle standard card (PDF# 52–1907) (Figure 1P). The XPS pattern of PRM‐siRNA (Figure 1Q) shows that PRM‐siRNA has the electronic orbital peak of ruthenium, which also confirms the successful modification of the ruthenium complex. In Figure 1R gel retardation experiments demonstrated that PR could effectively wrap siRNA (complete siRNA loading weight ratio: 1:1, 2.5:1, 5:1, 10:1, 15:1, and 20:1, agarose gel retardation analysis; from left to right).

### 2.2 PRM‐siRNA Inhibited Aβ Aggregation

Thioflavin T (ThT) analysis was used to assess the ability of nanoparticles to prevent the assembly of Aβ fibrils or to disassemble Aβ_42_ fibrils. The interaction of ThT with amyloid aggregates results in a significantly enhanced fluorescence signal. As shown in Figure 1S, PRM‐siRNA effectively depolymerized Aβ aggregates after coincubation with Aβ aggregates, and the depolymerization effect was concentration dependent. Figure 1T shows that the ThT fluorescence intensity decreased as the incubation time changed, indicating a decrease in the number of Aβ aggregates. Considering the key role of Cu^2+^ in AD, the therapeutic significance of Cu^2+^ capture by PRM‐siRNA was investigated. After adding PRM‐siRNA to the Cu^2+^ solution, the concentration of residual Cu^2+^ in the supernatant decreased significantly within 5 min, indicating that PRM‐siRNA NPs could effectively capture Cu^2+^, which was measured by atomic absorption spectrometry (Figure 1U). To further observe whether the nanoparticles could inhibit the aggregation of β‐amyloid, we incubated the Aβ protein monomer with different nanoparticles. Figure 1V shows that the fluorescence intensity was significantly lower after PRM‐siRNA treatment than after Aβ treatment alone, indicating that PRM‐siRNA has an inhibitory effect on the formation of Aβ fibrils. In addition, we found that NIR light excitation significantly enhanced the effect of PRM‐siRNA on the formation of Aβ fibrils. As seen in Figure 1W,X, compared with that of PR, the soluble Aβ content of the supernatant of the reaction system was significantly increased and the average particle size of Aβ aggregates was reduced, indicating that PRM‐siRNA has a stronger ability to depolymerize Aβ aggregates, which is similar to the results monitored by ThT experiments.

TEM analysis was used to determine the inhibitory effect of the PRM‐siRNA on the morphology of assembled Aβ_42_ oligomers. When Aβ protein and Cu^2+^ exist together, because Cu^2+^ [8] will induce the aggregation of Aβ, after 48 h, a large amount of Aβ protein aggregation occurs (Figure S2A). After adding NPs to the preincubated Aβ fibers, the fibrosis of the Aβ protein in the NP group was significantly reduced. PR reduced most of the long fibers to short fibers, and in the PRM‐siRNA group, Aβ protein binds to NPs and becomes spherical aggregates (shown in red circles in Figure S2B). Therefore, the TEM data confirmed that PRM‐siRNA is involved in the inhibition of Aβ fibrosis.

**FIGURE 2:**
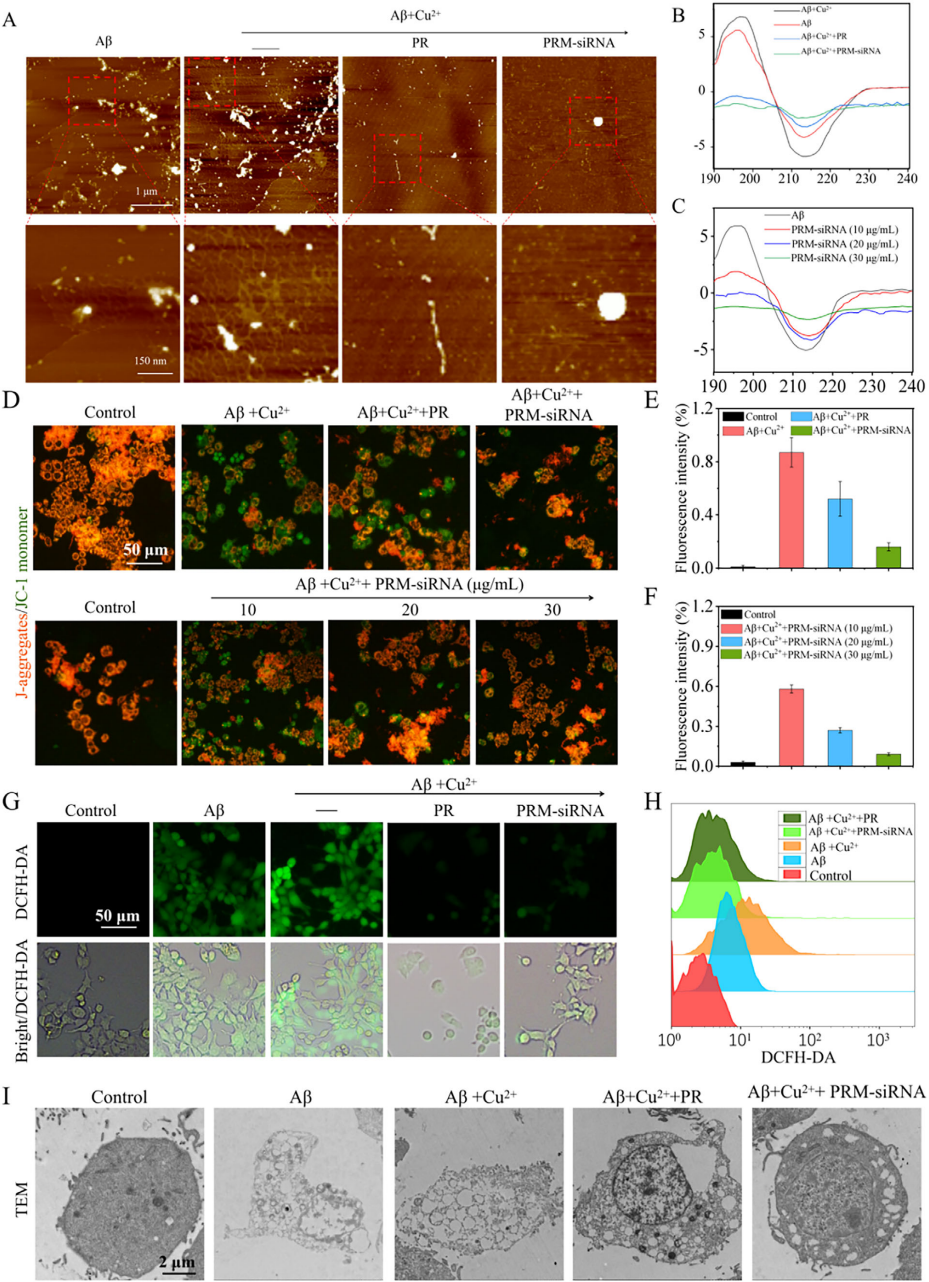
PRM‐siRNA reduced the Aβ fibrosis in cells. (A) AFM analysis of Aβ aggregation in the presence or absence of PR and PRM‐siRNA. Aβ was incubated with different nanomaterials for 48 h, the morphological changes of Aβ aggregates were observed using an AFM. The red box indicates the region where the Aβ aggregates are amplified. CD spectroscopy analysis of Aβ fibril secondary structure: (B) Changes in Aβ secondary structure after 48 h incubation at 37°C in the presence or absence of PR and PRM‐siRNA were monitored by CD spectroscopy. (C) Changes in Aβ secondary structure after 48 h incubation at 37°C in the presence of different concentrations of PRM‐siRNA. (D) Fluorescence images and (E,F) fluorescence intensity of the effect on mitochondrial membrane potential of PC‐12 cells in the presence of mixtures of Aβ, Aβ+Cu^2+^ and Aβ/NPs and different concentrations of PRM‐siRNA. Green fluorescence indicates JC‐1 monomer, which indicates low mitochondrial membrane potential. Red fluorescence indicates JC‐1 aggregation, indicating a high mitochondrial membrane potential. (G) Fluorescence image of ROS generation in PC‐12 cells in the presence of a mixture of Aβ, Aβ+Cu^2+^ and Aβ/NPs with DCFH‐DA as a fluorescent probe. (H) ROS changes in PC12 cells detected by flow cytometry. (I) TEM images of PC12 cells after 12 h of treatment with different NPs. The blank group was treated with PBS. Each value represents the mean standard deviation (*n* = 3).

The Aβ monomer sample was incubated with different NPs for 48 h to observe whether there was aggregate formation by an atomic force microscope. Figure 2A shows that after 48 h of incubation of Aβ samples, the formation of branched fibers was observed (shown as red boxes in the figure). With the addition of Cu^2+^, Aβ formed a large number of branched fibers. On the other hand, when Aβ, Cu^2+^, and PR were incubated for 48 h, only short fibers were formed, and long fibers were almost invisible. Only spherical aggregates were observed in the Aβ, Cu^2+^, and PRM‐siRNA groups. The AFM experiment showed that NPs effectively inhibited the aggregation and fibrosis of Aβ amyloid, which was consistent with the results of ThT fluorescence analysis and TEM analysis.

### 2.3 Change in the Secondary Conformation of Aβ

Studies have shown that the formation of Aβ aggregates and fibers usually involves the transformation of the α‐helical structure into β‐sheets, and the presence of metal ions will accelerate the aggregation of Aβ in solution [3–5, 9]. Therefore, we used circular dichroism spectroscopy (CD spectroscopy) to study the changes in protein secondary structure to verify the inhibitory effect of NPs on Aβ protein aggregation. The proportion of β‐sheets and CD signal strength are directly related to the number of Aβ fibers. Figure 2B shows the changes in the CD spectrum of Aβ after 48 h of incubation with different NPs. When Aβ alone exists, there is a positive peak at 195 nm and a negative peak at 218 nm, which indicates that there is a β‐sheet structure and Aβ has fibers. When Aβ was incubated with Cu^2+^, the intensity of the positive and negative peaks at 195 nm increased, and the intensity of the negative peak at 218 nm increased, indicating the existence of abundant fibrotic protein. For the mixture of Aβ + Cu^2+^ + NPs, the intensity of the positive peak at 195 nm decreases, and the intensity of the negative peak at 218 nm decreases. Moreover, compared with PR, the peak intensity of PRM‐siRNA is lower, indicating that Aβ fibrosis is lower, and it can be seen from Figure 2C that the change in the secondary conformation of Aβ is similar to that of PRM‐siRNA concentration is also related. These results provide important support for the capacity of PRM‐siRNA NPs to disintegrate Aβ_42_ fibrils in vivo.

### 2.4 PRM‐siRNA Reduced the Aβ Fibrosis in Cells

To further evaluate Aβ fibrosis in PC12 cells, fluorescence detection was performed in the presence of Cu^2+^, PR or PRM‐siRNA with 0.05% ThT and DAPI (1 µg mL^−1^) solution. Figure S3A illustrated that the Aβ group produced stronger green fluorescence, and the Aβ + Cu^2+^ group had stronger fluorescence than that of Aβ group, indicating that more Aβ fibers were produced. The PR group had a lower green fluorescence intensity, indicating that NPs reduced the aggregation of Aβ fibers. The green fluorescence of PRM‐siRNA group was the lowest, indicating that PRM‐siRNA greatly reduced the fibrosis of Aβ monomers. This conclusion was further confirmed by the change in relative fluorescence intensity (Figure S3B). These results indicated that PRM‐siRNA prevented Aβ monomers from forming Aβ fibrils in vitro.

### 2.5 PRM‐siRNA Inhibited Aβ‐Induced Mitochondrial Dysfunction

To explore the cell death mechanism, JC‐1 staining was used to examine the cell apoptosis. As shown in Figure S3C and Figure 2D, cells exposed to Aβ or Cu^2+^ alone both showed significant apoptosis and loss of mitochondrial membrane potential. Combined treatment of Aβ and Cu^2+^ showed enhanced cell apoptosis and the loss of mitochondrial membrane potential. However, PRM‐siRNA co‐treatment significantly inhibited Aβ‐induced apoptosis and the loss of mitochondrial membrane potential in PC12 cells. The results indicated that PRM‐siRNA inhibited the production of Aβ fibrils and attenuated Aβ fibrils‐induced cytotoxicity and apoptosis by inhibiting mitochondrial dysfunction. The relative fluorescence intensity further confirmed this conclusion (Figure S3D, Figure 2E,F). Taken together, these results revealed that PRM‐siRNA had the potential to inhibit the production of Aβ fibrils and attenuate Aβ fibrils‐induced cytotoxicity and cell apoptosis by inhibiting mitochondrial dysfunction.

### 2.6 PRM‐siRNA Blocked Aβ‐Induced ROS Production in PC12 Cells

To investigate whether the toxicity of Aβ is mediated by ROS induced by Aβ fibrils, the fluorescent probe DCFH‐DA was used to detect changes in ROS levels. The production of ROS in PC12 cells was detected by the fluorescence intensity of DCF. Figure 2G shows the fluorescence image of ROS generation in PC12 cells in the presence of a mixture of Aβ, Aβ + Cu^2+^, and Aβ/NPs with DCFH‐DA as a fluorescent probe. Figure 2H shows that in the Aβ and Aβ + Cu^2+^ groups, the stronger fluorescence intensity indicates that more ROS are produced by Aβ fibrils, and higher ROS levels will cause cell toxicity. The fluorescence intensity of the PRM and PRM‐siRNA groups was very weak, and the ROS level was very low, indicating that PRM‐siRNA can reduce the increase in Aβ‐induced ROS levels, thereby reducing the ROS‐mediated increase in cytotoxicity. From Figure 2H, the ROS intensity detected by flow cytometry can also be seen in this result. The ROS levels of the Aβ and Aβ + Cu^2+^ groups were higher than those of the other groups. These data indicate that PR and PRM‐siRNA can reduce the amount of ROS, thereby reducing the cytotoxicity induced by Aβ fibrils.

### 2.7 PRM‐siRNA Improved the Cell Ultrastructure in Aβ‐Treated Cells

To observe the effect of Aβ fibrils on cell integrity, transmission electron microscopy was used to observe ultrathin sections of PC12 cells treated with NPs. Ultrathin cell sections can be used to observe changes in the internal structure of cells due to their ultrathin characteristics. Significant changes in the internal structure of cells exposed to Aβ or Aβ+Cu^2+^ were significantly observed, such as, the cells disintegrated, the cytoplasm leaked from the cell, the structure of the nucleus was ambiguous, the organelles were not obvious, so many cell fragments around, and some hollow parts were observed (Figure 2I). The PRM‐siRNA group cell morphology and the blank group showed no obvious change that the nucleus was visible, and the cytoplasm was obvious. The above results indicated that Aβ damaged the nuclear structure and integrity of the cell, but PRM‐siRNA improved the cell ultrastructure in Aβ‐treated cells.

### 2.8 PRM‐siRNA Treatment Inhibited Aβ‐Induced Cytotoxicity and Apoptosis

**FIGURE 3:**
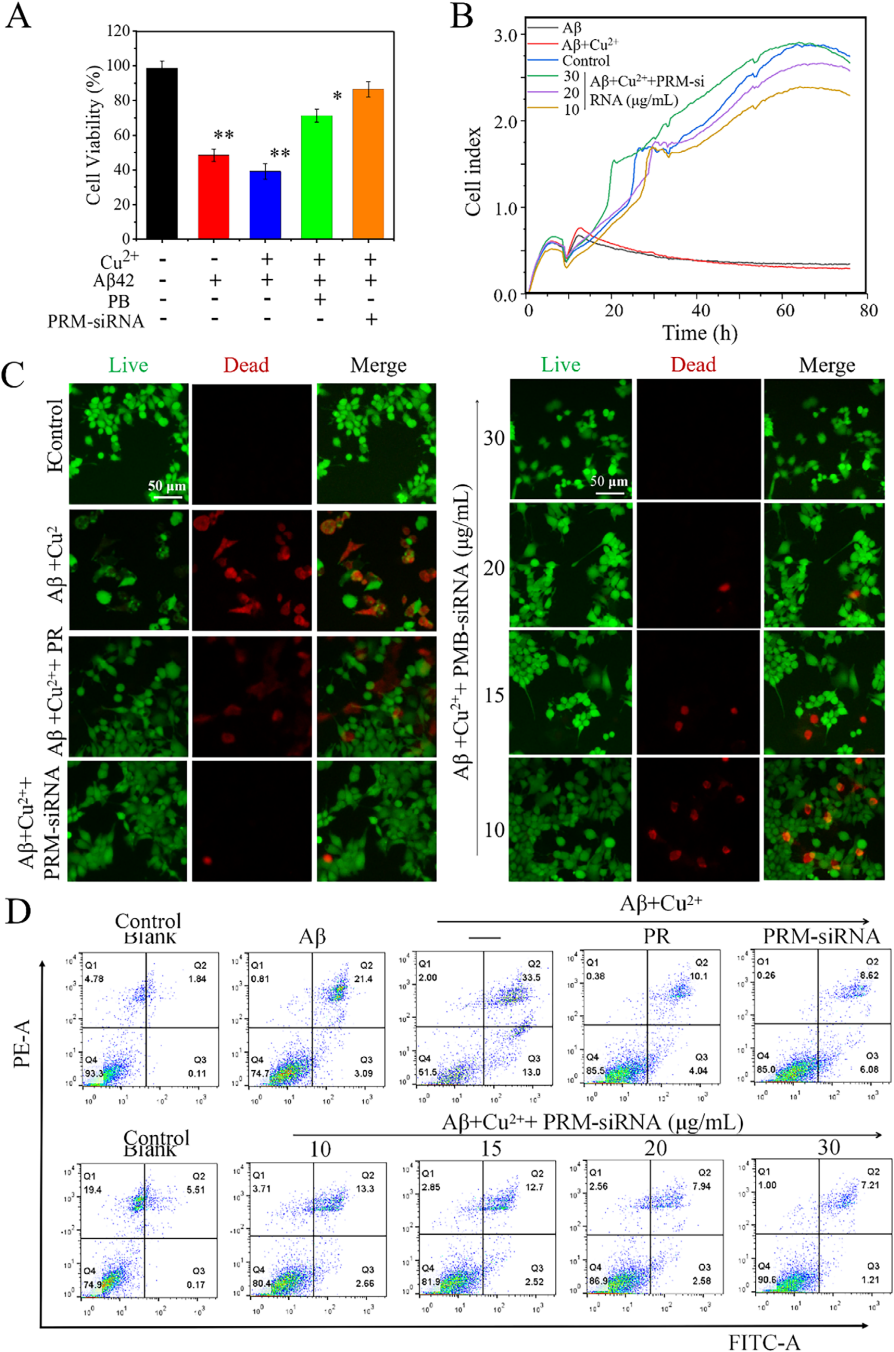
PRM‐siRNA inhibited Aβ‐induced apoptosis PC12 cells. (A) Effect of Aβ monomers on PC12 cell viability in the absence and presence of Cu^2+^, PB and PRM‐siRNA. (*p < 0.05, **p < 0.01, compared to control groups). (B) Dynamic monitoring of the cytotoxicity using the xCELLigence RTCA system. (C) Live/Dead cells assay. Cells were treated with Aβ fibers and/or different concentrations of PRM‐siRNA for 24 h, and then fluorescent microscopic images of PC12 cells were obtained by LIVE‐DEAD staining. (D) Cell apoptosis detection. Cells after treatment were analyzed by Annexin V/PI staining with flow cytometer. Each value represents the mean standard deviation (*n* = 3).

Aβ fibers showed severe toxicity to nerve cells [8, 45, 46], which will affect the normal growth of cells and lead to symptoms of AD. To examine the cytotoxicity of Aβ fibrils, CCK‐8 assay was employed to detect the Aβ‐induced cells cytotoxicity in PC12 cells. As shown in Figure 3A, the CCK‐8 results clearly showed that Aβ fibrils treatment caused obvious cytotoxicity, and combined treatment of Aβ and Cu^2+^ showed enhanced cytotoxicity. Cell viability dynamically monitored by xCELLigence RTCA system further confirmed the cytotoxicity (Figure 3B). Moreover, LIVE/DEAD assay by calcein (AM) and propidium iodide (PI) staining further confirmed that Aβ or Aβ + Cu^2+^ induced significant PC12 cells death (Figure 3C). Furthermore, annexin V/PI co‐staining by flow cytometry further confirmed that Aβ or Aβ + Cu^2+^ induced significant PC12 cells apoptosis (Figure 3D). However, PRM‐siRNA co‐treatment significantly inhibited Aβ‐induced cytotoxicity and apoptosis in PC12 cells. Meanwhile, PRM‐siRNA co‐treatment significantly improved the PC12 cells morphology in Aβ‐treated cells. As shown in Figure S4, cells exposed to Aβ or Cu^2+^ alone both showed light cell morphological changes, such as a decrease of cell number, cell shrinkage, and loss of neurites connection. Combined treatment of Aβ and Cu^2+^ showed enhanced cell morphological damage. However, PRM‐siRNA co‐treatment significantly improved cell morphology in Aβ‐treated cells, which further confirmed the protective effects.

### 2.9 PRM‐siRNA Suppressed Aβ‐Induced Neurotoxicity by Inhibiting ROS‐Mediated Oxidative Damage and Regulating Bcl‐2 Family in Rat Primary Neurons

**FIGURE 4:**
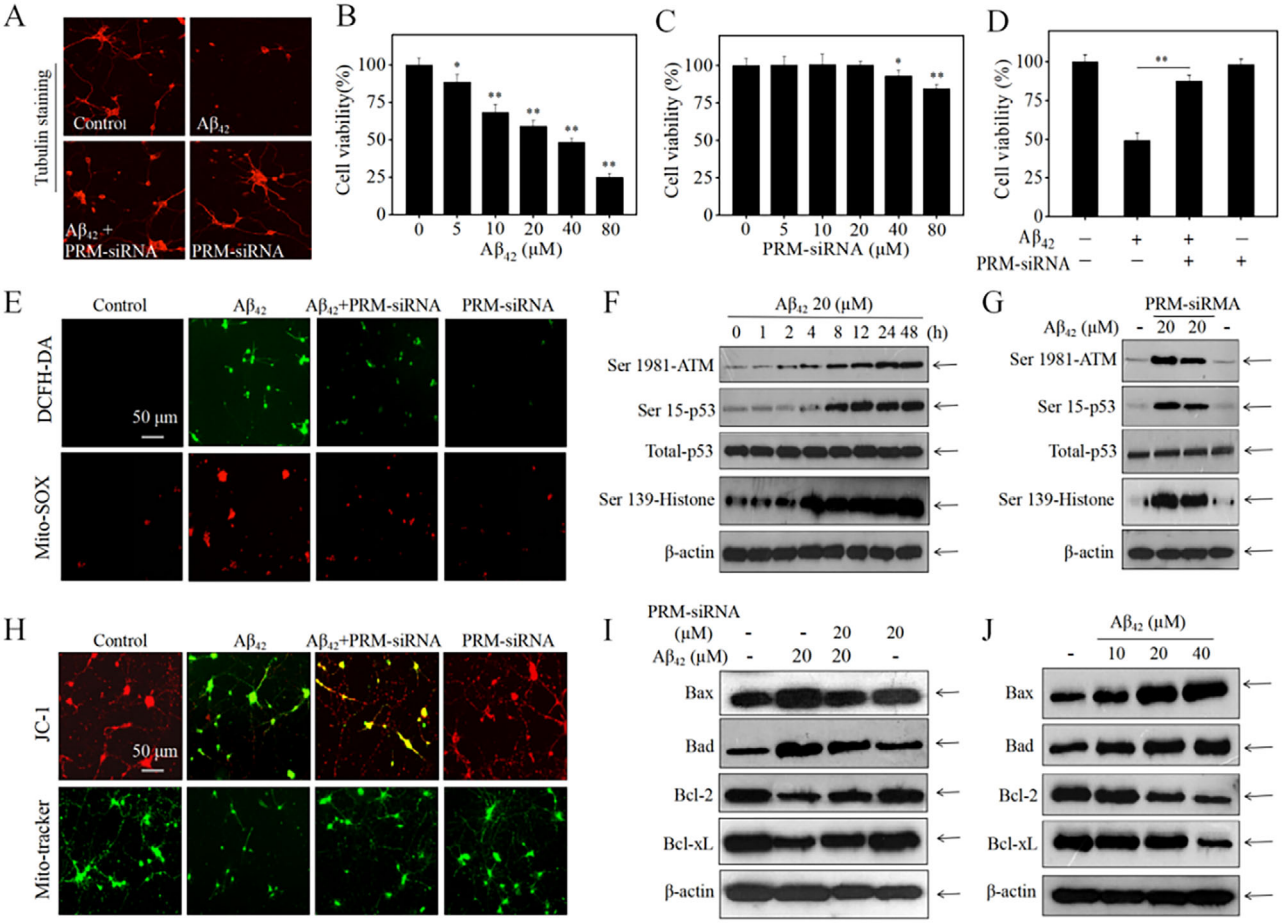
PRM‐siRNA attenuated primary neurons apoptosis by inhibiting ROS‐mediated oxidative damage, mitochondrial dysfunction and regulating Bcl‐2 family. (A) Tubulin staining for neural morphological detection. Primary neurons were treated with 20 µg mL^−1^ of PRM‐siRNA and 20 µM Aβ_42_ for 48 h, changes in neuronal morphology were observed by Tubulin under fluorescence microscopy. (B) Aβ_42_‐induced neuronal toxicity. (C) Cytotoxicity of PRM‐siRNA towards neurons. (D) PRM‐siRNA inhibited Aβ_42_‐induced neuronal toxicity. (E) PRM‐siRNA inhibited Aβ_42_‐induced ROS generation. (F) Aβ_42_ induced DNA damage in a time‐dependent manner. Protein expression was examined by western blotting. (G) PRM‐siRNA attenuated Aβ_42_‐induced DNA damage. (H) PRM‐siRNA inhibited Aβ_42_‐induced neuronal mitochondrial dysfunction. (I) PRM‐siRNA attenuated Aβ_42_‐induced abnormal expression of Bcl‐2 family proteins. (J) Aβ_42_ dose‐dependently affected the expression of Bcl‐2 family proteins. All experiments were done at least three times.

The protective effects and underlying mechanism of PRM‐siRNA against Aβ were further explored in rat primary neurons. As shown in Figure 4A–D, CCK‐8 result indicated that PRM‐siRNA (5–20 µM) showed no significant cytotoxicity toward primary neurons. Aβ treatment significantly inhibited neural viability with a dose‐dependent manner. However, PRM‐siRNA co‐treatment effectively inhibited Aβ‐induced neural cytotoxicity, and the neural morphological improvement by tubulin staining further confirmed this protective effect. To further explore the molecular, Mito‐SOX and DCFH‐DA probes were used to detect the ROS accumulation. Mito‐tracker and JC‐1 probes were used to detect the mitochondrial dysfunction. The results indicated that Aβ treatment caused obvious generation of superoxide anions and ROS (Figure 4E), and triggered the loss of mitochondrial membrane potential and mitochondrial fragmentation (Figure 4H). Moreover, Aβ treatment subsequently caused oxidative damage. As shown in Figure 4F, Aβ treatment time‐dependently activated the phosphorylation of ATM, p53, and histones, indicating that Aβ caused significant DNA damage. Figure 4J showed that Aβ treatment also caused Bcl‐2 family expression imbalance. However, PRM‐siRNA co‐treatment significantly attenuated Aβ‐induced neurotoxicity, inhibited mitochondrial dysfunction and ROS generation, and suppressed Bcl‐2 family expression imbalance and oxidative damage. Taken together, these results indicated that PRM‐siRNA had the potential to suppress Aβ‐induced neurotoxicity by inhibiting ROS‐mediated oxidative damage and regulating Bcl‐2 family in rat primary neurons.

### 2.10 PRM‐siRNA Under NIR Showed Enhanced BBB Permeability

**FIGURE 5:**
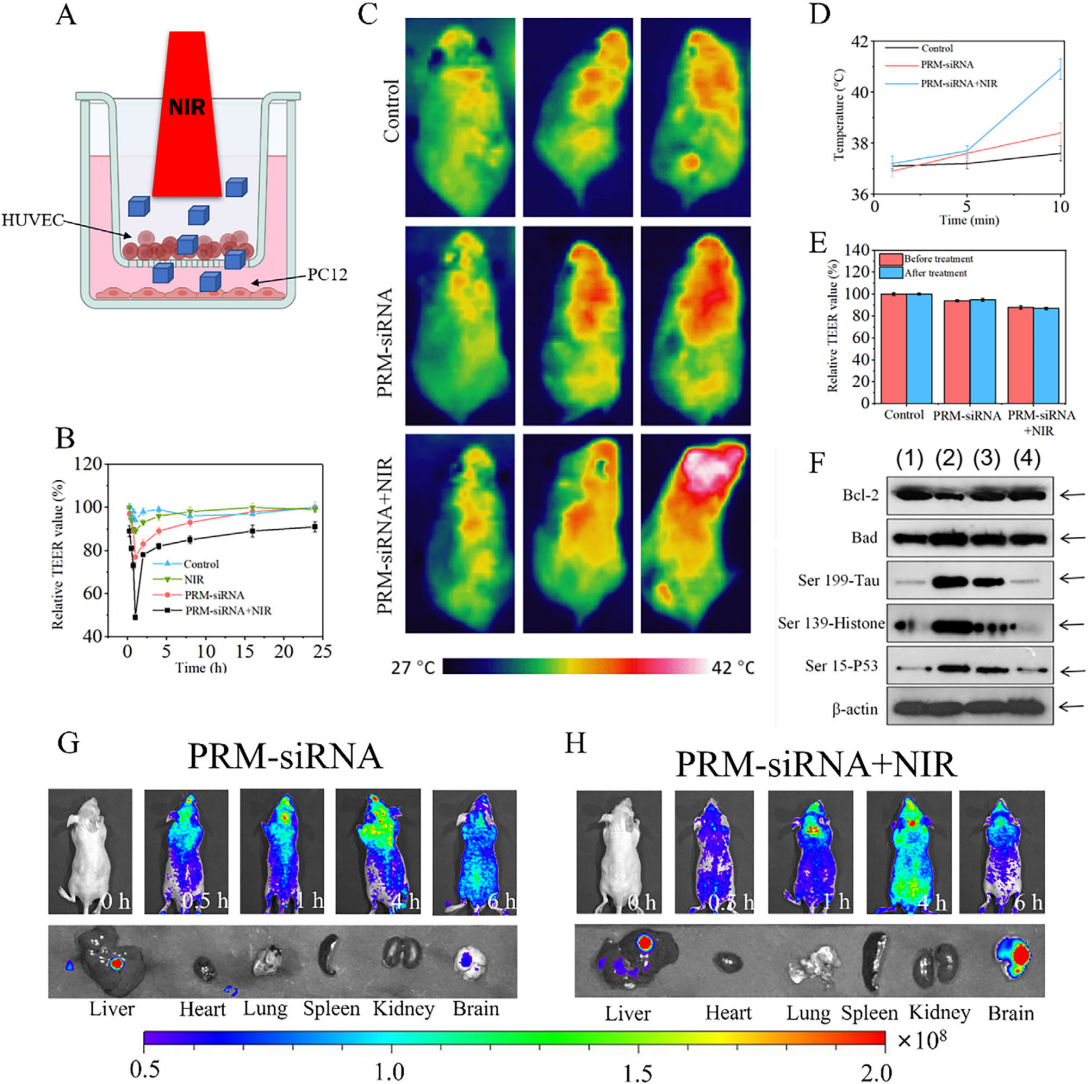
PRM‐siRNA under NIR showed enhanced BBB permeability. (A) Schematic diagram of the principle of Transwell or TEER (time‐dependent transendothelial cell resistance) measurement to evaluate the permeability of co‐cultured cells by measuring the top (inner chamber) and basal (outer chamber) chamber electrode resistance. The inner chamber cells are human umbilical vein endothelial cells and the outer chamber cells are PC12 cells. (B) TEER values change after the addition of NPs. (C) Photothermal imaging. NPs drug was injected through tail vein and photothermal imaging was obtained by NIR irradiation. (D) Changes of head temperature in mice with NIR irradiation time. (E) Changes in TEER values before and after NPs treatment. (F) WB images of mouse brain tissue treated with different nanoparticles. (1) WT. (2) AD. (3) AD + PRM‐siRNA. (4) AD + PRM‐siRNA + NIR. Fluorescence distribution of (G) PRM‐siRNA and (H) PRM‐siRNA + NIR in mice. All experiments were done at least three times.

To evaluate the in vivo protective mechanism, the BBB permeability of PRM‐siRNA was firstly explored by establishing an in vitro BBB model with human umbilical vein endothelial cells (HUVECs) using a Trans‐well microplate. As shown in Figure 5A,B, the transendothelial layer resistance (TEER) value of PRM‐siRNA under NIR irradiation showed a significant decrease in a time‐dependent manner, indicating that PRM‐siRNA by photothermal effect under NIR irradiation instantaneously opened the BBB for enhanced drug delivery. The in vivo TEM results indicated that PRM‐siRNA under NIR irradiation successfully crossed the BBB and accumulated in the brain parenchyma of APP/PS1 mice (Figure S5). The in vivo photothermal imaging in mice further confirmed the enhanced BBB drug delivery. As shown in Figure 5C,D, mice after injection with PRM‐siRNA by tail vein showed a significant increase of brain temperature under NIR irradiation, which was higher than that of PRM‐siRNA alone. The TEER value before and after the injection of PRM‐siRNA further confirmed that PRM‐siRNA, by photothermal effect under NIR irradiation, instantaneously opened the BBB and enhanced the BBB permeability for drug delivery (Figure 5E). The in vivo real‐time fluorescence imaging in APP/PS1 double transgenic mice further confirmed the enhanced BBB permeability. As shown in Figure 5G,H, PRM‐siRNA‐treated APP/PS1 mice after NIR irradiation (808 nm, 0.2 W cm^−2^) showed obvious enhanced red fluorescence in the mice heads in a time‐dependent manner, which was higher than that of mice treated with PRM‐siRNA alone (without NIR irradiation). The bright red fluorescence of the detached brain further confirmed this conclusion. PRM‐siRNA can bind Aβ and show red fluorescence, and these results clearly validated that PRM‐siRNA opened the BBB and accumulated in mice brains. Taken together, these results above all revealed that PRM‐siRNA under NIR irradiation had the potential to instantaneously open the BBB and enhance the BBB permeability for drug delivery.

### 2.11 PRM‐siRNA Alleviated Neural Loss, Neurofibrillary Tangles and Activation of Astrocytes and Microglial Cells in APP/PS1 Mice

**FIGURE 6:**
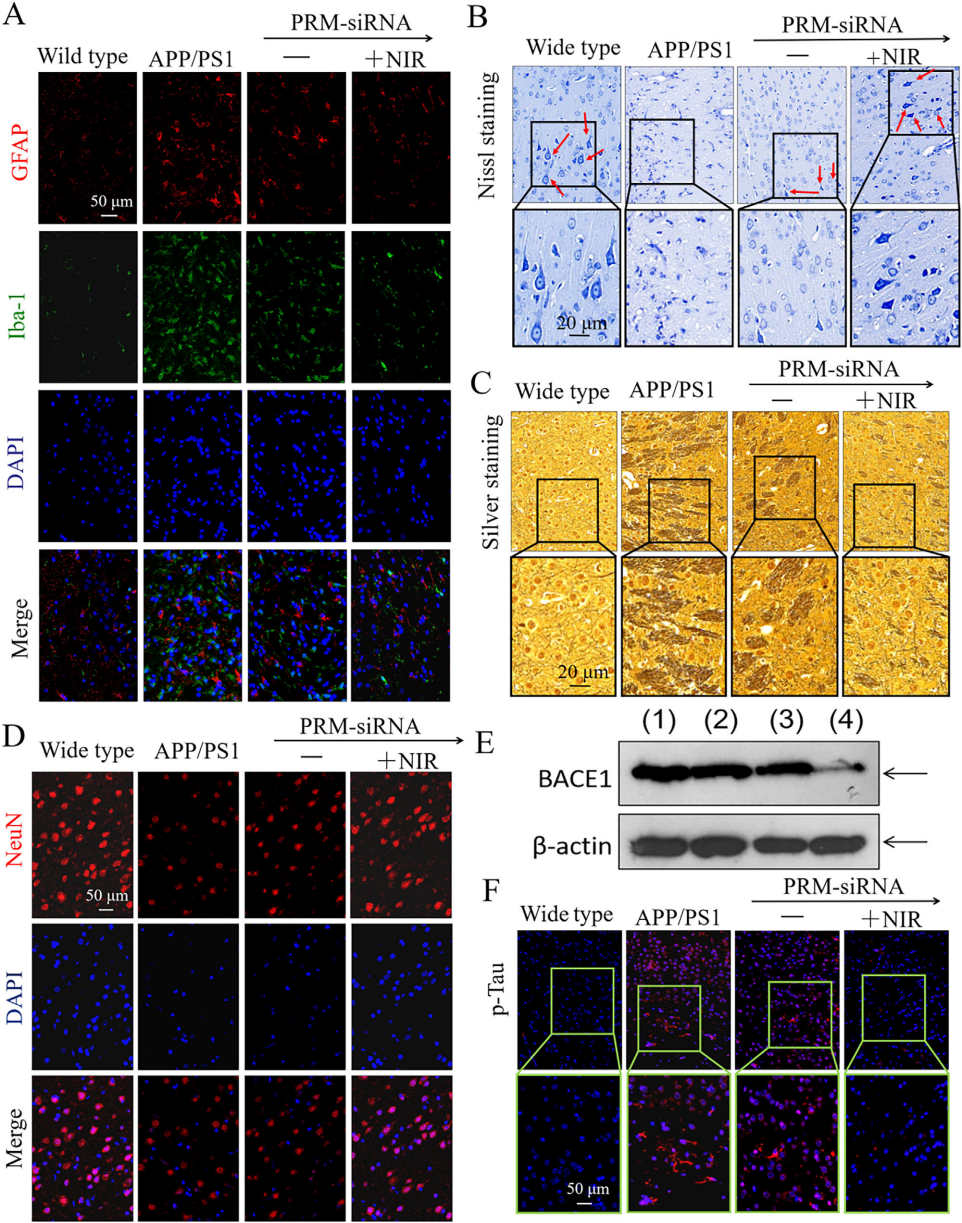
PRM‐siRNA alleviated neural loss, neurofibrillary tangles and activation of astrocytes and microglial cells in APP/PS1 mice. (A) PRM‐siRNA inhibited APP/PS1 mice activation of astrocytes and microglial cells. GFAP and IBA‐1 staining was conducted in mice brain tissue. (B) Nissl staining. (C) Silver staining for neurofibrillary tangles. (D) NeuN‐DAPI staining for neural death. (E) BACE1 expression in APP/PS1 mice. (F) P‐Tau immunofluorescence staining. All experiments were at least done three times.

To evaluate the in vivo protective mechanism, several brain tissue staining was employed to explore the neural loss, neurofibrillary tangles, and activation of astrocytes and microglial cells in APP/PS1 mice. First, Aβ staining indicated that PRM‐siRNA under NIR irradiation significantly inhibited Aβ deposition in the hippocampus of APP/PS1 mice (Figure S6). Iba‐1 and GFAP staining results indicated that APP/PS1 mice showed significant activation of astrocytes and microglia (Figure 6A). NeuN and Nissl staining indicated that APP/PS1 mice showed significant loss of neuron number and Nissl bodies (Figure 6B,D). Sliver staining results suggested that the brain of APP/PS1 mice showed neurofibrillary tangles (Figure 6C). Mechanism investigation suggested that the brain of APP/PS1 mice showed significant BACE1 decrease (Figure 6E) and tau hyperphosphorylation (Figure 6F). Moreover, Bcl‐2 family imbalance, Tau hyperphosphorylation, and oxidative damage (Ser139‐histone and Ser15‐p53) detected by western blotting were all found in APP/PS1 mice brains (Figure 5F). However, PRM‐siRNA under NIR irradiation significantly inhibited the loss of neuron and Nissl bodies, attenuated the activation of astrocytes and microglia, and blocked the neurofibrillary tangles and tau hyperphosphorylation. Mechanism study revealed that PRM‐siRNA under NIR irradiation significantly inhibited BACE1 expression, attenuated tau hyperphosphorylation and oxidative damage (Ser139‐histone and Ser15‐p53), and balanced the Bcl‐2 family expression. The statistics results further confirmed these protective effects (Figure S7A–F). Taken together, these results above all suggested that PRM‐siRNA under NIR irradiation alleviated neural loss, neurofibrillary tangles, and activation of astrocytes and microglial cells in APP/PS1 mice by inhibiting tau hyperphosphorylation and BACE1 expression, attenuating oxidative damage, and regulating Bcl‐2 family expression.

### 2.12 PRM‐siRNA Improved APP/PS1 Mice Learning and Memory

**FIGURE 7:**
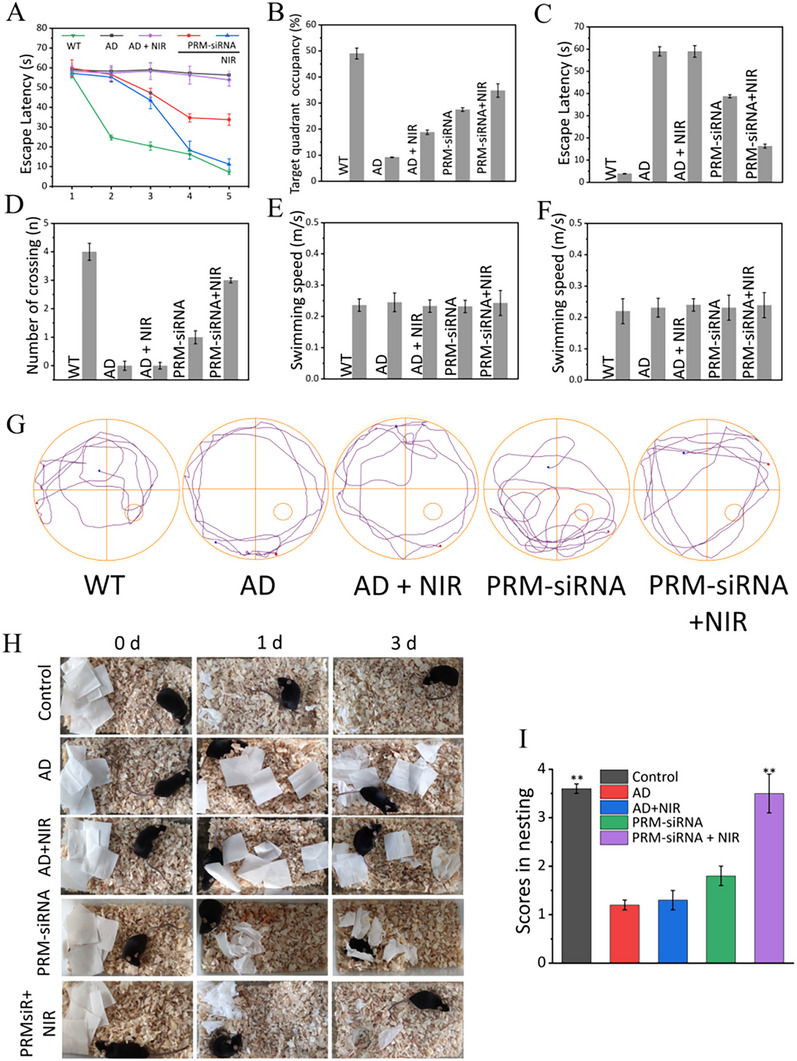
PRM‐siRNA improved the learning and memory of APP/PS1 mice. MWM (Morris water maze) experiment. (A) The APP/PS1 mice of different groups found the change in the escape latency of the hidden platform during the five‐day training period. (B) In the test period without a hidden platform, the effect of nanomaterials on the percentage of APP/PS1 mice target quadrant occupancy. (C) The effect of nanomaterials on the escape latency of APP/PS1 mice during the test period without a hidden platform. (D) During the test period without a hidden platform, the effect of nanomaterials on the number of times APP/PS1 mice cross the target platform. (E) During the training period, the effect of nanomaterials on the average swimming speed of APP/PS1 mice. (F) During the test period, the effect of nanomaterials on the average swimming speed of APP/PS1 mice. Each value represents the mean standard deviation (*n* = 3). (G) Changes in the path of mice during the test period after removing the hidden platform. On the 6th day, a spatial probe test was carried out. The path of the mouse in the water maze was recorded in 60 s. (H) and (I) The nesting behavior of AD mice was quantified, and WT mice of the same age were used as controls. Representative images from 0 to 3 days. The data are presented as mean S.E. **p < 0.01.

Morris water maze was employed to examine the improvement of APP/PS1 mice learning and memory. After a 5‐day training, all APP/PS1 mice were given a water maze test. As shown in Figure 7A–G, APP/PS1 mice showed slow swim speed, high escape latency, more times crossing the target platform and short residence time, indicating that APP/PS1 mice showed significant dysfunction in learning and memory ability. The mice nesting experiment further confirmed these observations and obtained similar results (Figure 7H,I). However, PRM‐siRNA under NIR irradiation significantly improved the mice's swim speed, escape latency, times crossing the target platform, and residence time, which were all better than that of AD + NIR and PRM‐siRNA. These results indicated PRM‐siRNA under NIR irradiation had the potential to improve APP/PS1 mice's learning and memory. The results of the Y‐maze likewise demonstrated similar conclusions in the ability to learn and remember. (Figure S8)

### 2.13 Biosafety Evaluation of PRM‐siRNA

The biological safety of PRM‐siRNA was evaluated by H&E staining of the main organs and blood biochemical indicators of mic. As shown in Figure S9A, the histological characteristics of the PR, PRM‐siRNA, PRM‐siRNA + NIR treatment groups and the blank group were similar, and there was no obvious damage or inflammation in any major organs. The hemolysis rate experiment was carried out on PRM‐siRNA, and it was found that no significant hemolysis occurred when the concentration of PRM‐siRNA increased to 100 µg mL^−1^ (Figure S9B). Blood biochemical examinations were performed on the mice, and the changes in the blood biochemical indicators of the mice after treatment were observed. As shown in Figure S9C–F, the levels of blood glucose, uric acid, cholesterol, and alanine aminotransferase (blood sugar, liver function, and kidney function) showed no significant changes in all groups. The results indicated that PRM‐siRNA has good biocompatibility and few side in vivo.

### 2.14 Gene Expression Analysis

**FIGURE 8:**
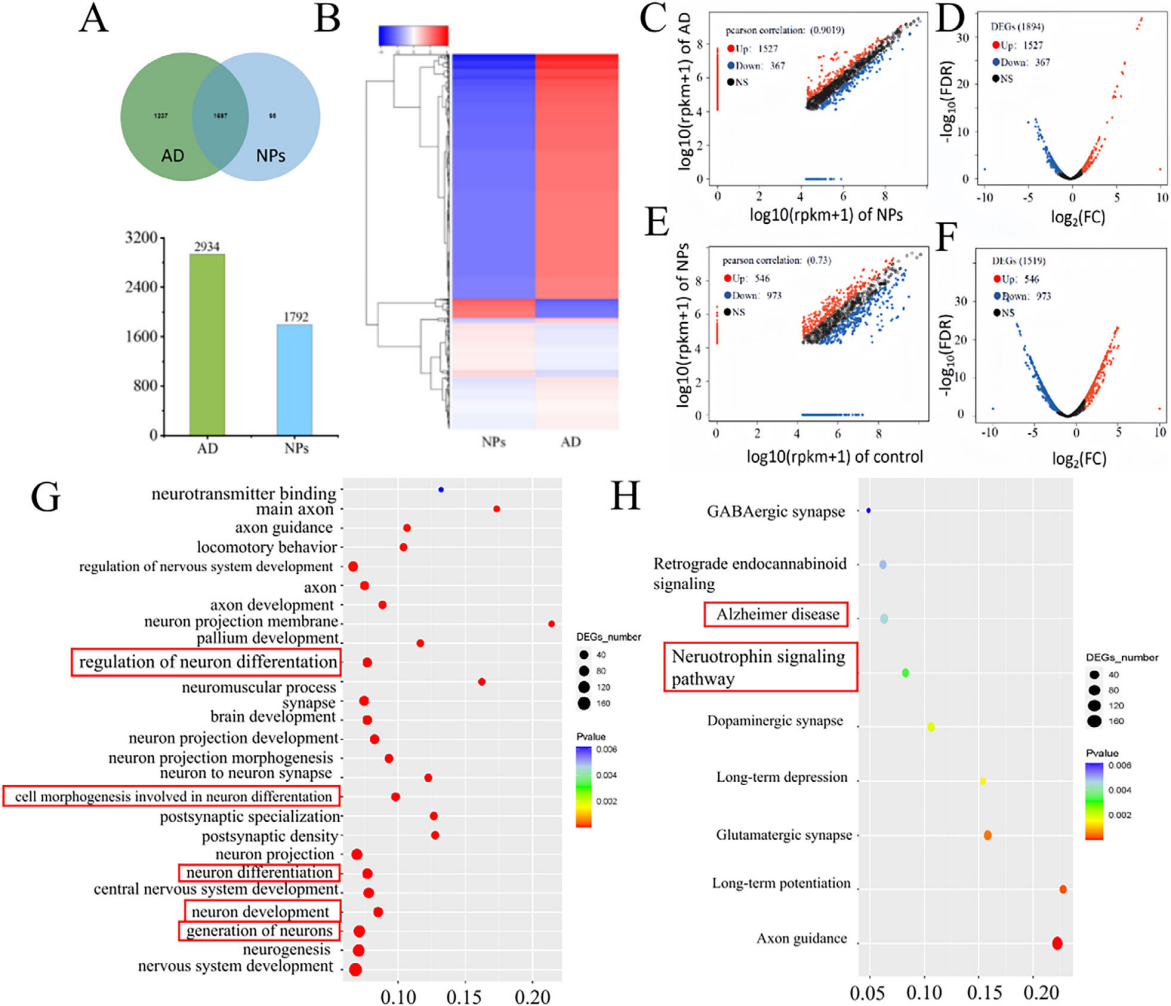
Differentially expressed genes analyzed in mouse brain tissue (AD mice vs. NPs treated AD mice, normal mice control). (A) The Venn diagram shows the overlap of differentially expressed genes obtained from sequencing genes after AD and NPs treatment. (B) Cluster analysis of differentially expressed genes. The red cluster indicates up‐regulated; the blue cluster indicates down‐regulated. (C) Scatter plot of Pearson correlation coefficient between NPs and AD groups. (D) Volcano plot of differentially expressed genes between NPs and AD groups. (E) Scatter plot of Pearson correlation coefficient between NPs and control groups. (F) Volcano plot of differentially expressed genes between NPs and control groups. (G) Statistical enrichment of differential expression genes in GO terms. (H) Statistical enrichment of differential expression genes in KEGG pathway.

Transcriptome changes between AD mice and PRM‐siRNA‐treated mice were analyzed. A Venn diagram showed overlapping sets of differentially expressed genes obtained for AD and NP treatment libraries after 24 h of exposure (Figure 8A). We compared the differential gene expression between the treatment group (PRM‐siRNA NPs) and the AD mouse group. Clustering analysis of the expression patterns of genes with significant differences (Figure 8B) can effectively find common points in expression between different genes, and the similarity of gene functions can be inferred based on the similarity in expression. Figure 8C shows the comparison between the AD group and the NP treatment group. Compared with the AD group, 1527 genes were up‐regulated, and 367 genes were down‐regulated in the NP treatment group. The volcano plot of the difference in gene expression between the AD group and the NP‐treated group also showed this situation (Figure 8D). However, the gene expression difference analysis of the NP treatment group versus the control (normal mice) showed that 546 genes were upregulated and 973 genes were downregulated (Figure. 8E). The volcano plot of gene expression differences between the NP‐treated group and the control group (normal mice) also showed changes in gene expression (Figure 8F). This result indicated that the difference in gene expression between the AD group and NP‐treated group showed that the expression of related genes in the AD group was more upregulated than that in the NP‐treated group, which may be because AD in mice affects gene expression. Compared with the control group (normal mice), the gene expression after NP treatment was upregulated and downregulated in a relatively normal range, which may be due to the treatment of NPs, which restored the AD mice to a relatively normal state. The specific gene expression differences were specifically analyzed in the subsequent GO and KEGG analyses.

### 2.15 GO Enrichment Analysis

Gene ontology (GO) is a database established by the Gene Ontology Consortium. Its purpose is to standardize the biological terms of genes and gene products in different databases and to define and describe the functions of genes and proteins. The GO function significant enrichment analysis of the differential genes can explain the functional enrichment of the differential genes and clarify the differences between samples at the gene function level. As shown in Figure S10 and Figure 8G, GO enrichment legend plot, GO analysis identified a total of eight terms related to cellular components, 17 terms for biological processes, and one term for molecular functions (AD versus NPs treatment group). Regarding biological process ontology, the most represented categories were regulation of neuron differentiation (GO: 0045664), cell morphogenesis involved in neuron differentiation (GO: 0048667), neuron differentiation (GO: 0030182), neuron development (GO: 0048666), and generation of neurons (GO: 0048699). The results showed that the neuronal differentiation and growth of AD mice were greatly improved after the AD mice were treated with PRM‐siRNA NPs. PRM‐siRNA NPs effectively improved neuronal development in AD mice.

### 2.16 Pathway Analysis by KEGG

KEGG (Kyoto Encyclopedia of Genes and Genomes) is a public database for genome deciphering. The study of biological pathways is critical to understanding and advancing genomics research. KEGG analysis revealed nine KEGG pathways. As shown in Figure S11 and Figure 8H, we selected pathways related to human diseases and organic systems for further analysis, and the AD versus NP genomic KEGG analysis showed that the AD group was significantly enriched in AD pathways compared with the PRM‐siRNA NP group. The results showed that after treatment with PRM‐siRNA NPs, the gene expression of mice could be affected to a certain extent to achieve the purpose of treating AD.

## 3 Discussion and Conclusion

Prussian blue nanoparticles were synthesized using hydrothermal synthesis, and ruthenium complexes ([Ru(bpy_)2_dppz]^2+^) were modified on Prussian blue nanoparticles by electrostatic adsorption. siRNA and metallothionein (MT) were modified on the nanoparticles using the cosolubilization method. Small interfering RNAs (siRNAs) combined with metallothionein‐modified ruthenium‐containing Prussian blue nanoparticles were synthesized (PRM‐siRNA). The morphological characteristics and particle size distribution of the nanoparticles were analyzed by transmission electron microscopy, scanning electron microscopy, and dynamic light scattering. PRM‐siRNA was a square nanoparticle of 60 ± 4 nm. Transmission electron microscopy, atomic force microscopy, CD spectroscopy, and ThT staining were used to observe the extent of Aβ fibrillation in vitro, and the results showed that PRM‐siRNA could inhibit the aggregation and disassembly of Aβ protofibrils in vitro.

At the cellular level, pheochromocytoma (PC12) cells and primary neuronal cells were cultured in vitro, and a neuronal injury model was constructed using Aβ. The experimental results showed that PRM‐siRNA inhibited Aβ‐induced neuronal activity in vitro in a dose‐dependent manner. PRM‐siRNA ameliorated mitochondrial dysfunction and inhibited apoptosis by regulating Bcl‐2 family genes. Immunofluorescence results showed that PRM‐siRNA inhibited Aβ‐induced ROS production and attenuated Aβ‐induced DNA damage, as evidenced by reduced phosphorylation levels of DNA damage markers. PRM‐siRNA attenuated Aβ‐induced oxidative damage and reduced Aβ protofibril‐induced cytotoxicity by inhibiting free radical production.

At the animal level, APP/PS1 double transgenic mice were used as the AD mouse model, and the results of mouse behavioral assays showed that PRM‐siRNA could effectively improve the learning and memory ability of AD mice. Immunofluorescence staining experiments showed that PRM‐siRNA effectively inhibited neuronal apoptosis, neurofibrillary tangles and reduced niche vesicles. The results of in vivo mechanistic studies showed that PRM‐siRNA significantly inhibited the activation of microglia and astrocytes and suppressed Tau protein phosphorylation in AD mice. Importantly, H&E staining results and blood biochemical indices showed good biocompatibility of the drug. Brain tissue gene expression sequencing results showed that PRM‐siRNA could function in AD mice and affect the expression of relevant genes in mice, thus improving the symptoms of AD mice. Importantly, PRM‐siRNA administration in vivo effectively improved APP/PS1 mice learning and memory by alleviating neural loss, neurofibrillary tangles, and activation of astrocytes and microglial cells in APP/PS1 mice by inhibiting BACE1 expression, oxidative damage, and tau phosphorylation. Taken together, our findings validated the rational design that BACE1 siRNA‐loaded Prussian blue nanocomplexes displayed enhanced BBB penetrability and synergy therapy for human AD.

## 4 Materials and Methods

### 4.1 Materials

Polyvinylpyrrolidone (PVP‐K30), [Ru(bpy)_2_dppz]^2+^, potassium ferricyanide (K_3_[Fe(CN)_6_]), and metallothionein (MT) were purchased from Sinopharm Chemical Reagent Co., Ltd. Metallothionein (MT) and siRNA‐BACE1 were purchased from Shanghai Jima Pharmaceutical Technology Co., Ltd. Thioflavin T (ThT), Cell Counting Kit‐8 (CCK‐8) assay kit, and AM/PI assay kit were purchased from Sigma. GPX4 mouse monoclonal antibody (1:5000; cat. no. sc‐166570; Santa Cruz Biotechnology, Inc.), GPX1 mouse monoclonal antibody (1:5000; cat. no. sc‐133160; Santa Cruz Biotechnology, Inc.), FTH1 mouse monoclonal antibody (1:1000; cat. no. SAB1405830; Sigma‐Aldrich), COX‐2 rabbit monoclonal antibody (1:200; cat. no. SAB5600153; Sigma‐Aldrich), ACSL4 mouse monoclonal antibody (1:1000; cat. no. sc‐365230; Santa Cruz Biotechnology, Inc.) and Phosphor(p)‐Tau (1:500; Ser 396 (ab32057)/199 (ab81268), Thr 205 (ab254410)/231(ab151559); Abcam). anti‐NeuN (1:500; cat. no. ab279296; Abcam), anti‐p‐Tau (1:500, Abcam), anti‐ACSL4 (1:200; cat.no.sc‐365230; Santa Cruz Biotechnology, Inc.), anti‐FTHI (1:200, Santa Cruz Biotechnology), anti‐COX‐2 (1:200, Sigma‐Aldrich), anti‐GFAP (1:500; cat. no. 130‐105‐140; DAKO) and anti‐Iba‐1 (1:500; cat. no. IRBAHUARAFIBA1C; Wako)

### 4.2 Synthesis of Prussian Blue Nanoparticles (PB NPs)

First, 1.5 g of polyvinylpyrrolidone (PVP‐K30) was added to 20 mL of ultrapure water, and the pH of the solution was adjusted to 2 with 6 mol L^−1^ hydrochloric acid and stirred for 5–10 min at room temperature to form a clear and transparent solution. Then, 45 mg of K_3_[Fe (CN)_6_] was added to the solution, and the solution was placed in a reaction kettle and heated at 80°C for 2 h. Subsequently, the solution was cooled to room temperature, the precipitate was centrifuged (13,000 g, 30 min) and washed after adding an appropriate amount of acetone, and the precipitate was dried under vacuum at 60°C for 10 h to obtain Prussian blue nanoparticles (PB NPs) [25].

### 4.3 Synthesis of PR NPs

PVP‐K30 (200 mg) and 20 mg of [Ru(bpy)_2_dppz]^2+^ were dissolved in 15 mL of ultrapure water, followed by the addition of 2 mL of formaldehyde and sufficient mixing. Two milligrams of PB NPs were added to the above mixed solution, and the reaction was continued at 150°C for 6 h. The precipitate was collected by centrifugation (13,000 g, 30 min) and washed three times with acetone, ethanol, and water. The ruthenium complex‐modified Prussian blue nanoparticles (PR NPs) were dried under vacuum at 60°C for 10 h to obtain [47].

### 4.4 Synthesis of PRM

Metallothionein (MT) peptides were modified on the surface of PR NPs using a cosolubilization method. In a typical synthesis, 5 mg PR NPs were dissolved in ultrapure water and then stirred at room temperature for 6 h (pH = 5.0) with the slow addition of 5 mL MT working solution (2.25 mg mL^−1^). Subsequently, the solution was centrifuged at 13,000 × *g* for 30 min to collect the precipitate, and the MT‐encapsulated PR NPs (PRM NPs) were obtained after washing the precipitate three times with ultrapure water.

### 4.5 Synthesis of PRM‐siRNA

PRM NPs were resuspended in RNase‐free ultrapure water. The 20 µM siRNA solution was mixed well with different weights of PRM NPs, and the siRNA was loaded onto the prepared nanoparticles by incubation for 20 min at room temperature to form nanocomposites (PRM‐siRNA NPs).

### 4.6 Characterization

The morphology of PB NPs and PRM‐siRNA NPs was observed by transmission electron microscopy (TEM, HT7700; Hitachi, Japan) and scanning electron microscopy (SEM, S‐4800; Hitachi; Japan). The crystal form of PRM‐siRNA NPs was detected by XRD, and the ruthenium elemental composition of the mesopores was determined by EDX (X‐Max N 150; Oxford; UK). The size distribution and zeta potential of the different nanoparticles were measured by using a Malvern Nanzo‐ZS nanoparticle size analyzer. Characterization was further performed by using Nicolet 6700 Fourier transform infrared spectroscopy (FT‐IR) and a Hitachi 3010 spectrophotometer for ultraviolet–visible (UV‒vis) absorption spectrum analysis. The ability of the polymer to concentrate siRNA was evaluated by agarose gel electrophoresis. Briefly, nanocomposites/siRNAs with different N/P ratios were electrophoresed on a 2% (w/v) agarose gel containing 0.1 µL mL^−1^ GelRed in 1×TBE running buffer at 120 V for 25 min. The sample was detected under a 302 nm ultraviolet illuminator and photographed using a fluorescence chemical imaging system.

### 4.7 Determination of Soluble Aβ Content

The degree of Aβ aggregation can be further determined by measuring the percentage of soluble Aβ in the supernatant of the reaction system. Aβ and different nanomaterials were incubated in PBS buffer for 48 h (37°C, 100 rpm). The solution was centrifuged at 20,000 rpm for 20 min and repeated three times. The concentration of soluble Aβ was determined by measuring the protein concentration of the supernatant with a BCA kit.

### 4.8 Real‐Time Cellular Analysis

Real‐time cellular analysis (RTCA, xCELLigence System, ACEA Biosciences Inc., Santiago, America) was used to examine the real‐time growth kinetics of PC12 cells treated with PRM‐siRNA. Briefly, 100 µL of medium was added to the e‐plate, and background readings were recorded, followed by inoculation of the cell suspension (100 µL) onto the e‐plate at a density of 2 × 10^4^ cells/well. The cell growth index was monitored every 10 min, and when the cells entered the logarithmic growth phase, the cells were washed once with fresh medium to remove any cell debris. Afterward, fresh medium containing different concentrations of PRM‐siRNA was added to each well, and the cell growth index was measured every 10 min for 75 h with RTCA.

### 4.9 Cell Viability Assay

PC12 cells (1 × 10^4^ cell/well) were added to a 96‐well plate and placed in a cell culture incubator for 24 h before adding nanoparticles and continuing to incubate for 12 h. Subsequently, 10 µL of CCK‐8 assay solution was added and incubated at 37°C for 2 h. The absorbance of each well was detected at 450 nm by a multifunctional enzyme marker. PC12 cells were grown in six‐well plates for 48 h. PR, PRM, and PRM‐siRNA (20 µg mL^−1^) were then added to the six‐well plates. After coculture for 24 h, the cells were stained with an AM/PI mixture for 5 min, observed and recorded by fluorescence microscopy. AM can stain all nuclei with green fluorescence, while propidium iodide (PI) can only stain damaged nuclei with red fluorescence.

### 4.10 Flow Cytometry Analysis

PC12 cells (5 × 10^5^ cells/well) were inoculated and grown in six‐well plates for 48 h, after which PR, PRM and PRM‐siRNA (20 µg mL^−1^) were added. After coculture for 24 h, cells were collected by digestion with EDTA‐free trypsin, followed by the addition of 5 µL of Annexin V‐EGFP and 5 µL of PI, and the reaction was performed for 20 min at room temperature, protected from light, and observed and detected by flow cytometry. Similarly, the cells collected after treatment were resuspended in 10 µM of 2',7'‐dichlorofluorescein diacetate (DCFH‐DA) and incubated at 37°C for 20 min. Afterward, cells were washed three times in the culture medium to completely remove DCFH‐DA that had not entered the cells, and flow cytometry was observed for detection.

### 4.11 Isolation and Culture of Rat Neuronal Cells

Newborn SD rats within one day of birth were selected, and brain tissues were obtained by aseptic craniotomy. The cerebral cortex tissue was separated, the cerebral cortex was repeatedly washed with dissecting fluid, and then the brain tissue was fully broken up and 5 mL of 0.25% trypsin was added. After digestion for 10 min, serum was added to terminate the digestion, and the cells were collected by centrifugation after filtering through a 200 mesh filter. The collected cells were resuspended in medium (80 mL DMEM high sugar medium, 10 mL fetal bovine serum, 10 mL calf serum, 1% penicillin and streptomycin, and 10 µg L^−1^ nerve growth factor) at 37°C and 5% CO_2_ for 24 h. After 4 days, 4 µg mL^−1^ agranulocyte was added to inhibit glial cells, and then the isolated neurons were used for subsequent experiments.

### 4.12 Western Blotting

After treating neurons with 10–40 µM Aβ for 48 h, cells were collected and lysed with cell lysis solution. The protein concentration was determined with a Bio‐Rad kit, and protein separation was performed by 8% SDS‐PAGE. The proteins were transferred to a polyvinylidene fluoride membrane, which was incubated with 5% bovine serum albumin (BSA) for 2 h at room temperature and then probed with primary antibodies (Bax, Bad, Bcl‐2, Bcl‐xL, β‐actin) overnight at 4°C. Blots were washed and incubated with appropriate secondary antibodies for 1 h at room temperature and finally detected with a chemiluminescence kit. Information about the antibodies we used is listed below: Mice were anesthetized with 1% sodium pentobarbital, dissected and their brain tissues (cortex) were removed in one piece. The brain tissues were ground and lysed, the protein levels determined for GPX4 mouse monoclonal antibody (1:5000; cat. no. sc‐166570; Santa Cruz Biotechnology, Inc.), GPX1 mouse monoclonal antibody (1:5000; cat. no. sc‐133160; Santa Cruz Biotechnology, Inc.), FTH1 mouse monoclonal antibody (1:1000; cat. no. SAB1405830; Sigma‐Aldrich), COX‐2 rabbit monoclonal antibody (1:200; cat. no. SAB5600153; Sigma‐Aldrich), ACSL4 mouse monoclonal antibody (1:1000; cat. no. sc‐365230; Santa Cruz Biotechnology, Inc.) and Phosphor(p)‐Tau (1:500; Ser 396 (ab32057)/199 (ab81268), Thr 205 (ab254410)/231(ab151559); Abcam) using Western blot analysis.

### 4.13 Observation of Neuronal Morphological Changes

Neuronal cells were cotreated with 20 µg mL^−1^ PRM‐siRNA and 20 µM Aβ for 48 h before being fixed with 3.7% formaldehyde solution for 20 min at room temperature. Subsequently, the cells were washed three times for 5 min each with PBS containing 0.1% Triton X‐100. Tubulin‐Tracker Red was diluted in PBS containing 2.5% bovine serum albumin (BSA) and 0.1% Triton X‐100 at a ratio of 1:100. Then, 100 µL of the diluted Tubulin‐tracker Red staining solution was added dropwise to each slide and incubated for 60 min at room temperature and protected from light. The slides were then washed three times with PBS containing 0.1% Triton X‐100 for 5 min each, after which they could be directly observed by fluorescence microscopy.

### 4.14 In Vitro Transport Studies of the BBB

To detect changes in BBB permeability, Transwell plates were used to construct a BBB model. Briefly, PC12 cells were seeded in the lower wall of Transwell plates (0.4 µm pore size) and cultured in DMEM containing 10% fetal bovine serum and 1% dual antibiotics for 2 days. Human umbilical vein endothelial cells (HUVECs) were seeded in the upper chamber of Transwell plates at a density of 1.0 × 10^5^ cells/well and cocultured with PC12 cells for 5 days. After culturing, dense cell layers were formed, and transendothelial layer resistance (TEER) was detected. Successful model construction was indicated when TEER was greater than 100 Ω. Subsequently, serum‐free medium and 20 µg mL^−1^ PRM‐siRNA were added to the upper chamber and irradiated by NIR (808 nm) for 10 min. TEER values were measured at 15 min, 30 min, 45 min, 60 min, 2 h, 4 h, 8 h, 16 h, and 24 h.

### 4.15 Animal Model

Clean healthy adult SD rats (male to female ratio, 2:1) were purchased from Jiangsu Lingfei Biotechnology Co., Ltd. After 1 week of adaptive feeding, they were caged together, and the occurrence time of female pubic occlusion was recorded to ensure sampling within 1 day of giving birth. Neonatal suckling mice within one day of birth were used for experiments to isolate neurons in the cerebral cortex.

C57 mice (8 months old, ∼30 g each) were purchased from Nanjing Meris Biotechnology Co., Ltd. for wild‐type group (WT) in animal experiments and drug biotoxicity assays. AD mice: All male wild‐type APP/PS1 mice (8 months old) were purchased from the Jiangsu Lingfei Technology Co., LTD. The APP/PS1 mice catalog is #004462. Food was removed for fasting 24 h before nanomedicine treatment. Two hundred microliters of 20 weight % (wt%) glucose was administered to all groups by intraperitoneal injection 30 min before nanomedicine injection. APP/PS1 and WT‐like mice injected with 200 µL of PBS were used as controls to demonstrate pathological dysfunction in AD mice. Dosing was started in 8‐month‐old mice and administered every other day for 2 months. All AD nanomedicine therapy groups were given 1 mg of siRNA equiv./kg diluted in 200 µL of PBS via caudal vein injection every 2 days. The group requiring NIR irradiation was irradiated with an 808 nm laser (0.2 W cm^−2^) for 5 min. The local temperature of the head was maintained at 41–43°C during irradiation, and behavioral experiments were performed 60 days after administration.

All animal experiments were carried out according to the plan approved by the Experimental Animal Center of Anhui Agricultural University (License No.: SYXK 2016‐007). Animal studies were carried out using the strict guidelines and in accordance with the Guidelines for The Care and Use of Experimental Animals in China. Two to five mice were housed in plastic cages with corncob bedding and free access to food and filtered water in a vivarium maintained on a 12‐h light/dark cycle at 22.0 ± 0.5°C.

### 4.16 Accumulation in Mouse Brain

APP/PS1 double transgenic mice were used as the AD mouse model. After injecting PRM‐siRNA into the tail vein, the mice were irradiated with NIR (808 nm, power 0.2 W cm^−2^) for 10 min on the head. In vivo fluorescence imaging was performed on the mice at 0, 0.5, 1, 4, and 6 h. The mice were subsequently dissected, and their major organs were fluorescently imaged.

Information about the antibodies we used in brain is listed below: brain sections were stained with primary anti‐NeuN (1:500; cat. no. ab279296; Abcam), anti‐p‐Tau (1:500, Abcam), anti‐ACSL4 (1:200; cat.no.sc‐365230; Santa Cruz Biotechnology, Inc.), anti‐FTHI (1:200, Santa Cruz Biotechnology), anti‐COX‐2 (1:200, Sigma‐Aldrich), anti‐GFAP (1:500; cat. no. 130‐105‐140; DAKO) and anti‐Iba‐1 (1:500; cat. no. IRBAHUARAFIBA1C; Wako) overnight at 4°C.

### 4.17 Behavioral Experiments

Treatments: APP/PS1 transgenic mice and WT mice of the same age were used for the experiments. All animals used for experiments were blindly assigned to treatment groups of 12 mice each to ensure experimental data accuracy. In the Morris water maze experiment, the pool of the water maze was 100 cm in diameter and 50 cm in height, and the water temperature was kept at 21 ± 2°C. The movement trajectories of the mice were recorded by a video tracking system. During the first 4 days of training, a hidden platform was placed in the middle of the target quadrant. During the 4‐day training period, mice were placed into the maze pool from different quadrants and trained to find the target platform, and the time for the mice to find the platform was the latency period. If the platform was not found after 60 s, the latency period was 59 s, and the mice were guided to find the platform area and stay there for a period of time. On day 5, the platform in the target quadrant was removed, the mice were tested for 60 s, and the number of times the mice crossed the original platform placement area and the time they stayed in the target quadrant were recorded and counted. Before the nesting experiment, six pieces of paper towels of the same size (5 cm × 5 cm) were placed in the bedding of each cage. The mice were placed in a separate cage, and the nesting condition of the mice was recorded after one day and three days. The index grading standard of the nesting experiment is: 0 = no pit and no paper towel; 1 = shallow pit without broken tissue; 2 = there are pits, shredded or whole paper towels; 3 = the pad pit surrounded by broken or whole paper towels, forming a cup‐shaped nest; and 4 = shredded paper towels form a spherical nest and cover the mouse.

### 4.18 Transcriptomic Analysis

Total RNA was extracted from the tissue using TRIzol Reagent according to the manufacturer's instructions (Invitrogen), and genomic DNA was removed using DNase I (TaKaRa). Then, RNA quality was determined using a 2100 Bioanalyzer (Agilent) and quantified using an ND‐2000 (NanoDrop Technologies). High‐quality RNA samples (OD260/280 = 1.8–2.2, OD260/230 ≥ 2.0, RIN ≥ 6.5, 28S:18S ≥ 1.0) were used to construct a sequencing library.

Small RNA‐seq libraries were prepared according to the TruSeq Small RNA sample preparation kit from Illumina (San Diego, CA). The total RNA was separated into different fragment sizes by polyacrylamide gel electrophoresis (PAGE). Small RNA fragments were selected preferentially 18–30 bp in size. Reverse transcription followed by PCR was used to create cDNA constructs based on the small RNA ligated with 3' and 5' adapters. The cDNA was purified and recovered by PAGE gel, and the product was dissolved in EB solution for subsequent labeling. Finally, library quality was assessed on the Agilent Bioanalyzer 2100 system. Paired‐end libraries were sequenced by Illumina NovaSeq 6000 sequencing (150 bp*2, Shanghai BIOZERON Co., Ltd).

The software was used to quantitatively analyze the expression levels of miRNAs in each sample for subsequent analysis of differential expression between different samples. After the read counts of miRNAs were obtained, the miRNA expression differences between samples were analyzed for multiple groups of samples (≥2 groups), and the differentially expressed miRNAs among the samples were identified. Target gene prediction software was used to predict the target genes of all known and newly predicted miRNAs, and GO and KEGG functional annotation and functional enrichment research were conducted on the target genes.

### 4.19 Statistical Analysis

All data was presented as mean ± SD. Statistical analysis was carried out by SPSS13.0 software. Statistical evaluation was processed by two‐tailed Student's *t*‐test between two groups. Statistical evaluation during three or more groups was one‐way ANOVA analysis followed by Dunnett's post‐hoc test or Tukey's post‐hoc test. Bars with different letters indicate statistically significant differences (*p *< 0.05). Bars with “*” or “**” represent the *p* < 0.05 or *p* < 0.01, respectively [48, 49, 50].

## Author Contributions

Xiaoyuan Ding, Yanyu Hu and Xiaotong Fu contributed equally to this work. Dongdong Sun, Xiaoyan Fu, and Cundong Fan designed the experiments. Chunxue Dai, Bangjia Yang, Xiaotong Feng, and Qile Song cooperated in completing in vitro experiments on PC12 cells and in vivo experiments within mice. Xiaoyuan Ding and Zekun Wang wrote the manuscript. Yanyu Hu, Xiaotong Feng, and Qile Song carried out the work of revising and refining the manuscript. Dongdong Sun, Xiaoyan Fu, and Cundong Fan conceived the conceptual ideas and supervised the study.

## Conflicts of Interest

The authors declare no conflicts of interest.

## Supporting information



Supporting Information

## Data Availability

The data that support the findings of this study are available from the corresponding author upon reasonable request.
