# Supplementary material for: Enhanced Blood‐Brain Barrier Penetrability of BACE1 SiRNA‐Loaded Prussian Blue Nanocomplexes for Alzheimer's Disease Synergy Therapy
Source: Exploration (Beijing). 2025 Mar 7;5(4):e20230178. doi: 10.1002/EXP.20230178 (PMC12380061; doi:10.1002/EXP.20230178)
Supplement: Supplementary file 1 — Supporting Information [file EXP2-5-e20230178-s001.docx]

Supporting Information

for

**Enhanced Blood-Brain Barrier Penetrability of BACE1 SiRNA-Loaded Prussian Blue Nanocomplexes for Alzheimer’s Disease Synergy Therapy**

Xiaoyuan Ding^1,3^, Yanyu Hu^1^, Xiaotong Feng^2^, Zekun Wang^1^, Qile Song^2^, Chunxue Dai^1^, Bangjia Yang^1^, Xiaoyan Fu^2*^, Dongdong Sun^1*^, Cundong Fan^2*^

^1^ School of Life Sciences, Anhui Agricultural University, Hefei 230036, China.

^2^ Shandong Key Laboratory of Brain Injury and Functional Rehabilitation, the Second Affiliated Hospital of Shandong First Medical University, Taian 271000, Shandong Province, China

^3^ College of Biotechnology and Pharmaceutical Engineering, West Anhui University, Luan 237012, China

Xiaoyuan Ding, Yanyu Hu and Xiaotong Feng contributed equally to this work.

* **Correspondence**

**Dongdong Sun**, School of Life Sciences, Anhui Agricultural University, Hefei 230036, China. Email: [sunddwj@ahau.edu.cn](mailto:sunddwj@ahau.edu.cn)

**Cundong Fan** and **Xiaoyan Fu**, Yingsheng Road 2, the Second Affiliated Hospital of Shandong First Medical University, Taian, Shandong Province, China. [cdfan@sdfmu.edu.cn](mailto:cdfan@sdfmu.edu.cn); [txyfu66@163.com](mailto:txyfu66@163.com)

**MATERIALS AND METHODS**

**Aβ fiber degradation experiment**

First, Aβ_42_ powder was dissolved in 1,1,1,3,3,3-hexafluoro-2-propanol (HFIP) to form a 1 mg mL^-1^ stock solution and stored at -20 °C. The peptides were dissolved in PBS buffer (10 mM, pH=7.4) by evaporating the solvent HFIP with a nitrogen stream before use and then filtered and diluted through a 0.22 μm filter membrane for experiments. In the Aβ fibril degradation experiments, the Aβ solution was first preincubated at 37 °C and 100 rpm for 3 days to allow complete formation of amyloid fibrils from Aβ. ThT fluorescence detection and circular dichroism (CD) spectroscopy (JASCO J-1500, Japan) were used to demonstrate that the Aβ monomer had been completely fibrillated under these conditions. Meanwhile, PR, PRM and PRM-siRNA were added to the Aβ fibril solution, and incubation was continued for 6-72 h. Samples were taken at intervals and observed by transmission electron microscopy.

**Cell culture**

Rat adrenal pheochromocytoma cells (PC12) were cultured in DMEM containing 10% horse serum, 5% fetal bovine serum and 1% streptomycin/penicillin, and the cells were placed in a 5% CO_2_ incubator at 37 °C.

**Apoptosis detection**

PC12 cells (5×10^5^ cells/well) were inoculated and grown in six-well plates for 48 h, and then PR, PRM and PRM-siRNA (20 μg mL^-1^) were added. After 24 h of incubation, the cells were fixed with 4.0% paraformaldehyde for 20 min and then permeabilized with 1% Triton X-100. Thereafter, the cells were placed in a TUNEL reaction mixture containing terminal deoxynucleotidyl transferase (TdT) and nucleotides for 1 h. The nuclei were further stained with DAPI, and PC12 cells were visualized by fluorescence microscopy. The decrease in mitochondrial membrane potential is one of the earliest events in the apoptotic cascade, and when the mitochondrial membrane potential collapses, apoptosis is irreversible. PC12 cells (5 × 10^5^ cells/well) were inoculated and grown in six-well plates for 48 h, followed by the addition of Aβ and PRM-siRNA (20 μg mL^-1^). After coculture for 24 h, the cells were stained with JC-1 (5,5’,6,6’-tetrachloro-1,1’,3,3’-tetraethylbenzimidazolcarbocyanine iodide) dye solution for 20 min, rinsed twice with PBS and then observed by fluorescence microscopy at 490 and 550 nm excitation wavelengths.

**Immunohistochemical and immunofluorescence staining of brain tissue**

After treatment, the mouse cerebral cortex was embedded in optimal cutting temperature (OCT) compound and immediately snap-frozen in liquid nitrogen. Serial frozen sections of 10 μm thickness were prepared using a frozen sectioning machine (HM525 NX, Thermo Fisher Scientific). All sections were stored at -20 °C until they were stained. Sections were washed in TBS buffer (0.15 M NaCl; 0.1 M Tris HCl, pH 7.5) for 20 min and then washed in TBS:BSA [containing 1% (w/v) BSA and 3% normal goat serum (NGS) incubated in TBS] for 30 min at room temperature to block nonspecific antibody binding. Sections were then incubated overnight with anti-NeuN, anti-GFAP and anti-Iba-1 antibodies. This was followed by treatment with HRP goat anti-rabbit (1:400, Abcam) for 1 h and visualization with a DAB kit (ZSGB-BIO, China). Finally, the nuclei were stained with DAPI and recorded by an Aperio VERSA section scanner (Leica, Germany).

**Y Maze Test**

To examine the spatial working memory capacity of mice, spontaneous alternation was tested using the Y-maze. The bottom and walls of the maze were made of white PVC. Three arms were positioned at an angle of 120° to each other and each arm was 30 cm × 7 cm × 18 cm (L × W × H). Mice were placed individually in the triangular center of the maze and allowed to explore freely for 8 min. The number of mice entering the arm entries and the number of correct alternations were recorded during this period to calculate the percentage of correct alternations. A correct alternation was defined as an entry that was different from the first two adjacent entries. The percentage of correct alternations was calculated as (number of correct alternations/ (total number of arm entries - 2)) × 100%. The total number of entries was used as a measure of motor activity.

**Results of mouse brain tissue sequencing data**

Small RNA sequencing was performed on the mouse brain (AD mice and NP-treated group). The quality and length of fragments were analyzed, linker sequences, contaminating sequences, and low-quality bases were removed, and clean small RNA sequences were obtained. In total, the AD group generated 28.405459 million raw reads with Q30 over 96.91%. After removing low-quality sequences (length < 35 bp; Q < 20), the retained clean reads totaled 28.333720 million. The siRNA-MT-PB-Ru NP group yielded 24.866828 million raw reads and 24.806266 million clean reads (Table S1). All error rates are low. Statistical analysis showed that the total number of reads in the sequencing samples was high, and the rate of high-quality reads was high. The results showed that the quality of the sequencing data is great.

**Table S1. Summary of the RNASeq Outcomes of AD and PRM-siRNA NP Samples**

| **Sample name** | **Raw Reads** | **Clean reads** | **Total Bases** | **Q20%** | **Q30%** |
| --- | --- | --- | --- | --- | --- |
| AD | 28405459 | 28333720 | 1416361716 | 99.23 | 96.91 |
| PRM-siRNA NPs | 24866828 | 24806266 | 1240033667 | 99.45 | 97.59 |


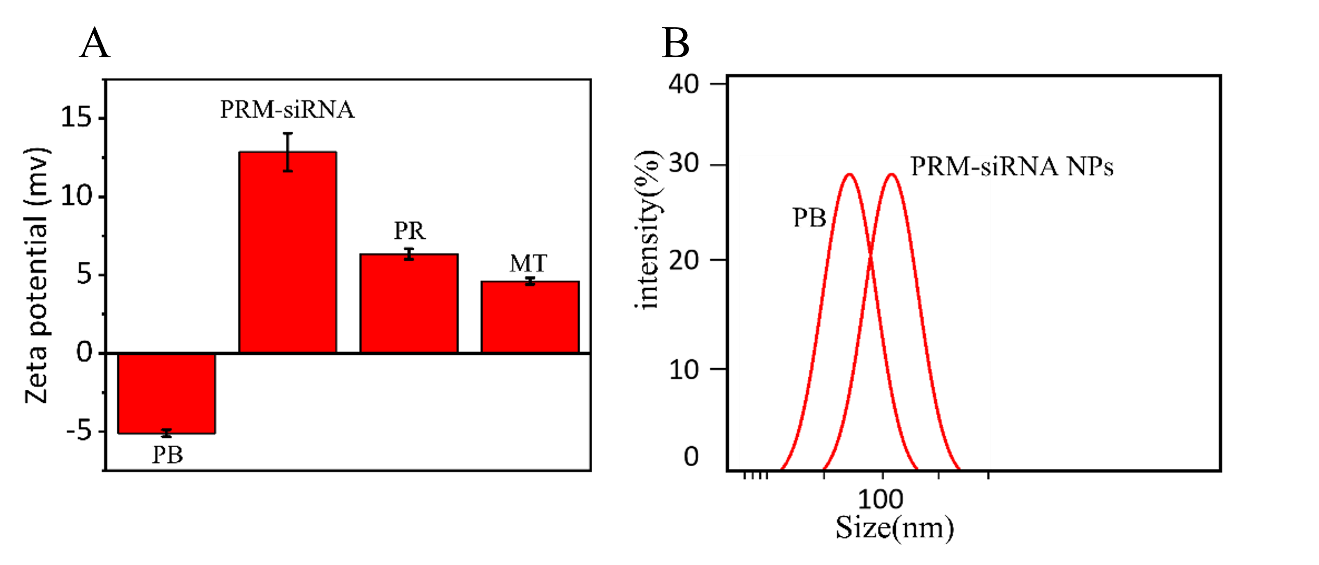


**Figure S1.** (A) Zeta potential (PB, PRM-siRNA, PR, MT). (B) Hydrodynamic size analysis (PB, PRM-siRNA)

**
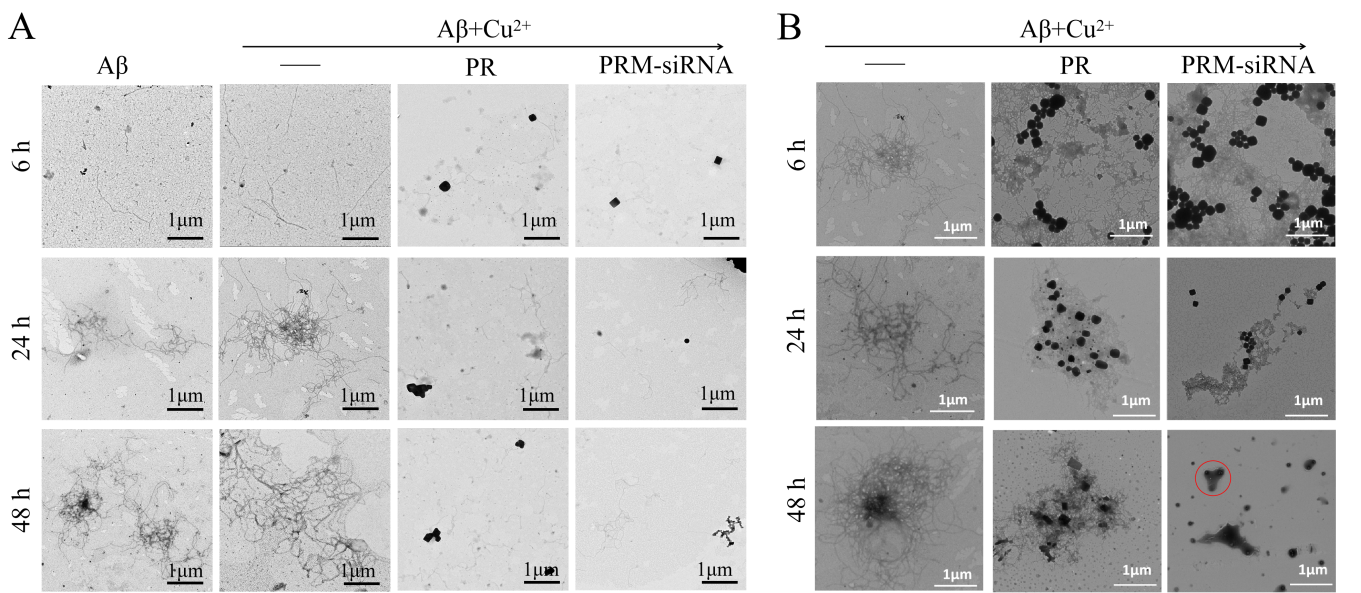
**

**Figure S2.TEM observation of the influence of NPs on Aβ fibers.** (A) TEM image of Aβ monomer and different nanoparticles after co-incubation for 6-48 h. (B) The decomposition of Aβ fibrils by PRM-siRNA. PRM-siRNA was added to the pre-incubated Aβ fibrils, incubated together for 0-48 h, and tested samples with TEM at different incubation times. The red circles indicate the formation of spherical aggregates.


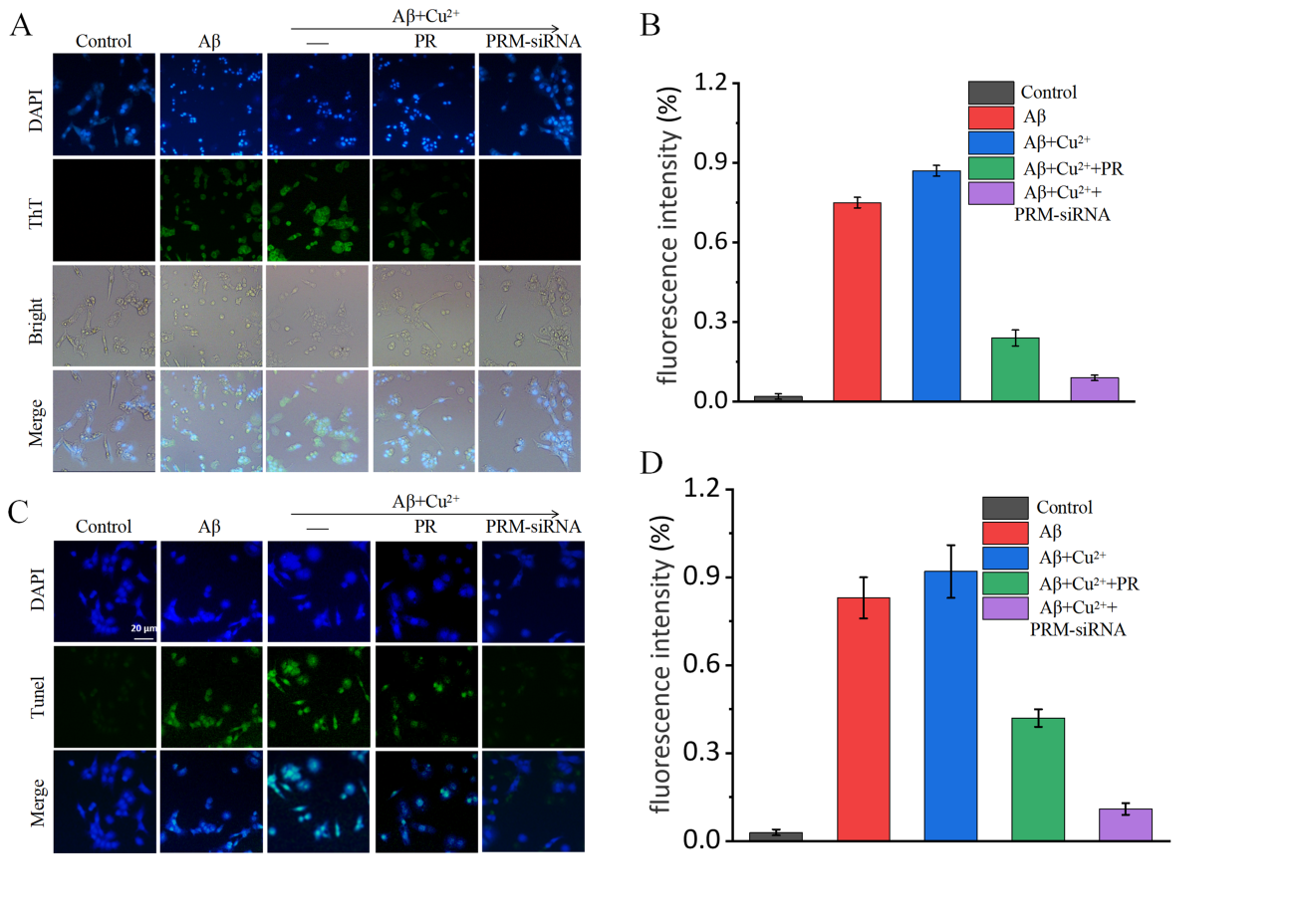


**Figure S3.** (A) Using DAPI and thioflavin T (ThT) as fluorescent probes, fluorescence images were generated by intracellular Aβ fibers in the presence of a mixture of Aβ, Aβ+Cu^2+^ and Aβ/NPs. ThT will combine with Aβ amyloid fibers to produce fluorescence, and the stronger the fluorescence, the more Aβ fibers. (B) Quantitative analysis of the level of Aβ amyloid fibrils presented by ThT fluorescence intensity. (C) Fluorescence images of DAPI (blue) and TUNEL (green) staining of apoptotic cells in the presence of Aβ, Aβ+Cu^2+^ and Aβ/NP mixtures. (D) Quantitative analysis of the level of apoptosis presented by TUNEL fluorescence intensity. Each value represents the mean standard deviation (n = 3).


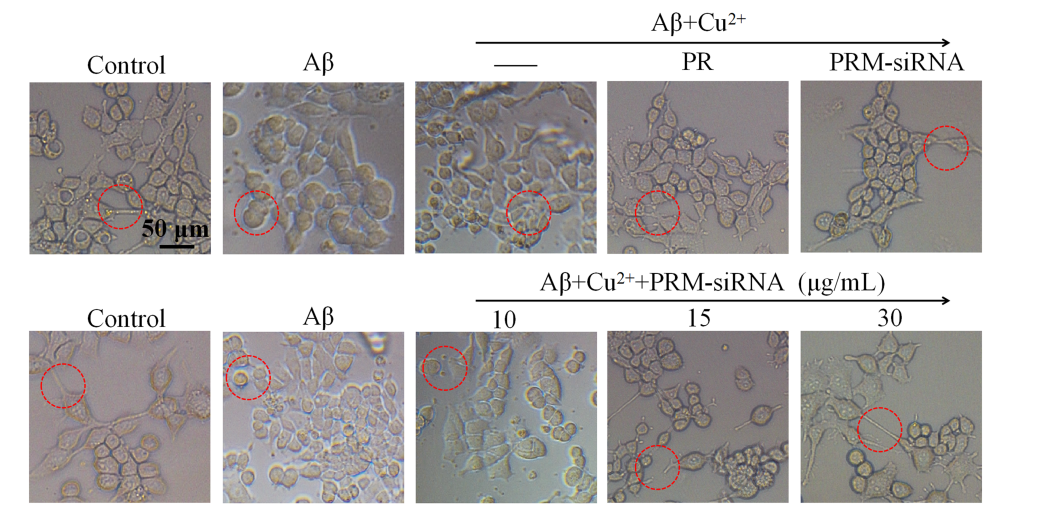


**Figure S4.** After treating the cells with Aβ fibers and different concentrations of PRM-siRNA for 24 h, the morphology of PC12 cells was observed under light microscopy, and the red circles indicate the changes in cell morphology.


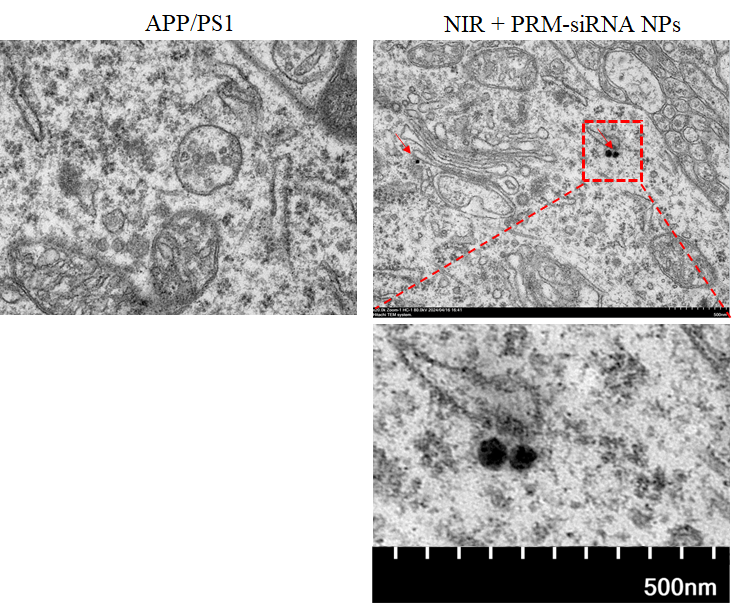


**Figure S5.** **PRM-siRNA under NIR irradiation successfully crossed the BBB and accumulated in in the brain parenchyma of APP/PS1 mice**. APP/PS1 mice were cut into sections, and PRM-siRNA accumulation in brain was detected by TEM.


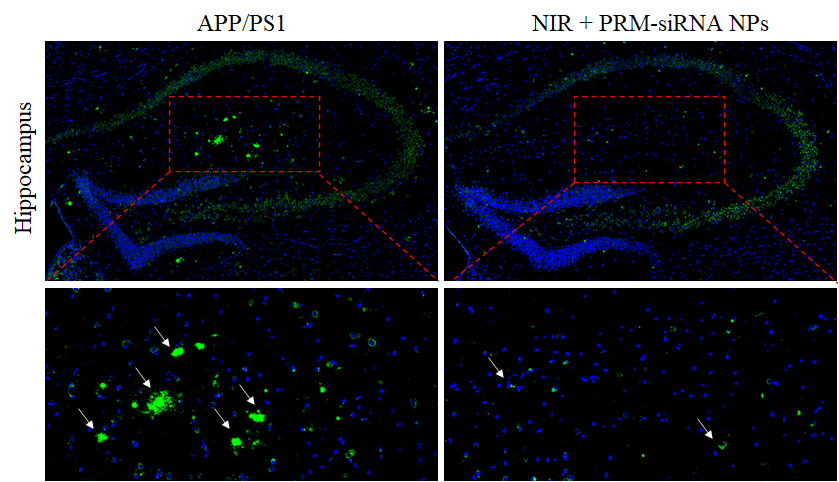


**Figure S6. PRM-siRNA under NIR irradiation significantly inhibited** **Aβ deposition in hippocampus of APP/PS1 mice.** Brain tissue from APP/PS1 mice were cut into 4 µM sections, and stained by anti-Aβ primary antibody, and the Aβ deposition in hippocampus of APP/PS1 mice was detected by immunofluorescence methods.


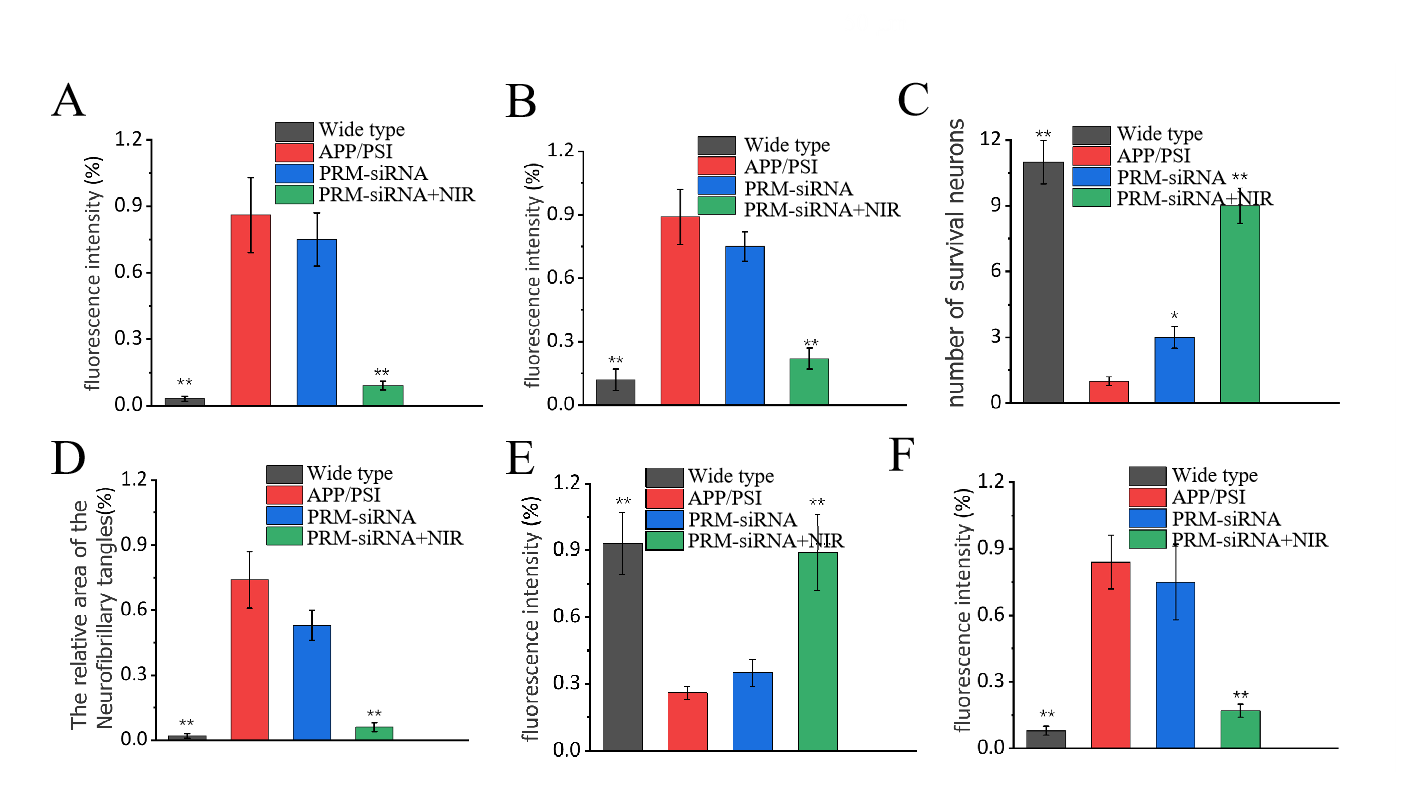


**Figure S7.** Quantitative analysis of brain tissue staining in APP/PS1 mice. (A) Relative fluorescence intensity analysis of GFAP. (B) Analysis of relative fluorescence intensity of IBA-1. (C) The number of Nissier vesicles in the Nissier stained pictures. (D) The relative areas of the nerve fiber tangles in this silver-stained image. (E) Relative fluorescence intensity analysis of NeuN. (F) Changes in fluorescence intensity in the immunofluorescence image. Data are presented as mean ± standard deviation (n = 3), ** p<0.01.* p<0.05.


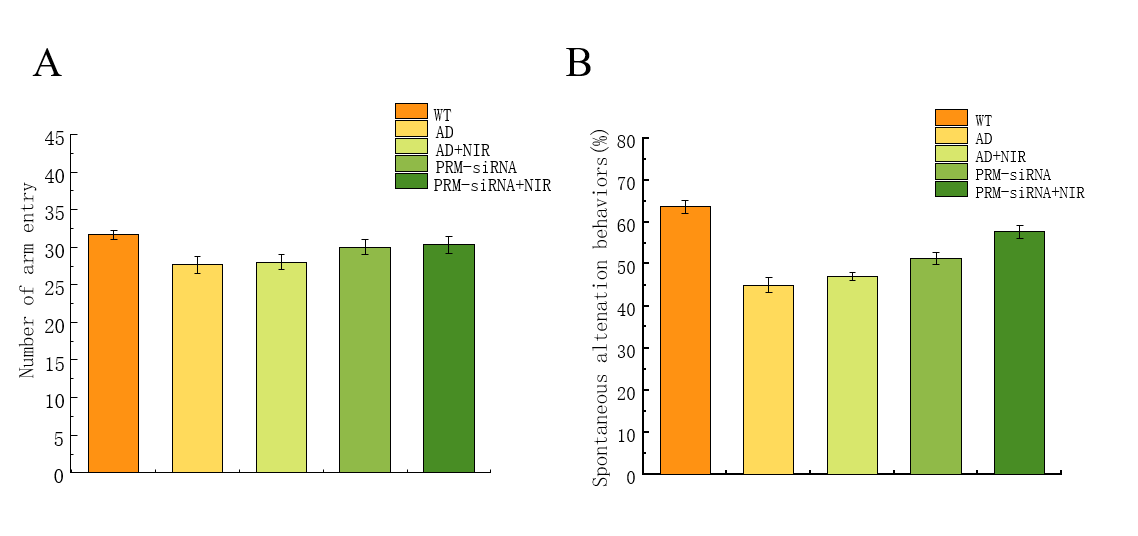


**Figure S8. PRM-siRNA improves working memory in the Y-maze task in AD mice.** (A) Total number of arm entries in all groups. (B) Alternation rate of spontaneous behaviors in mice in all groups. Data are presented as mean ± standard deviation (n = 3).


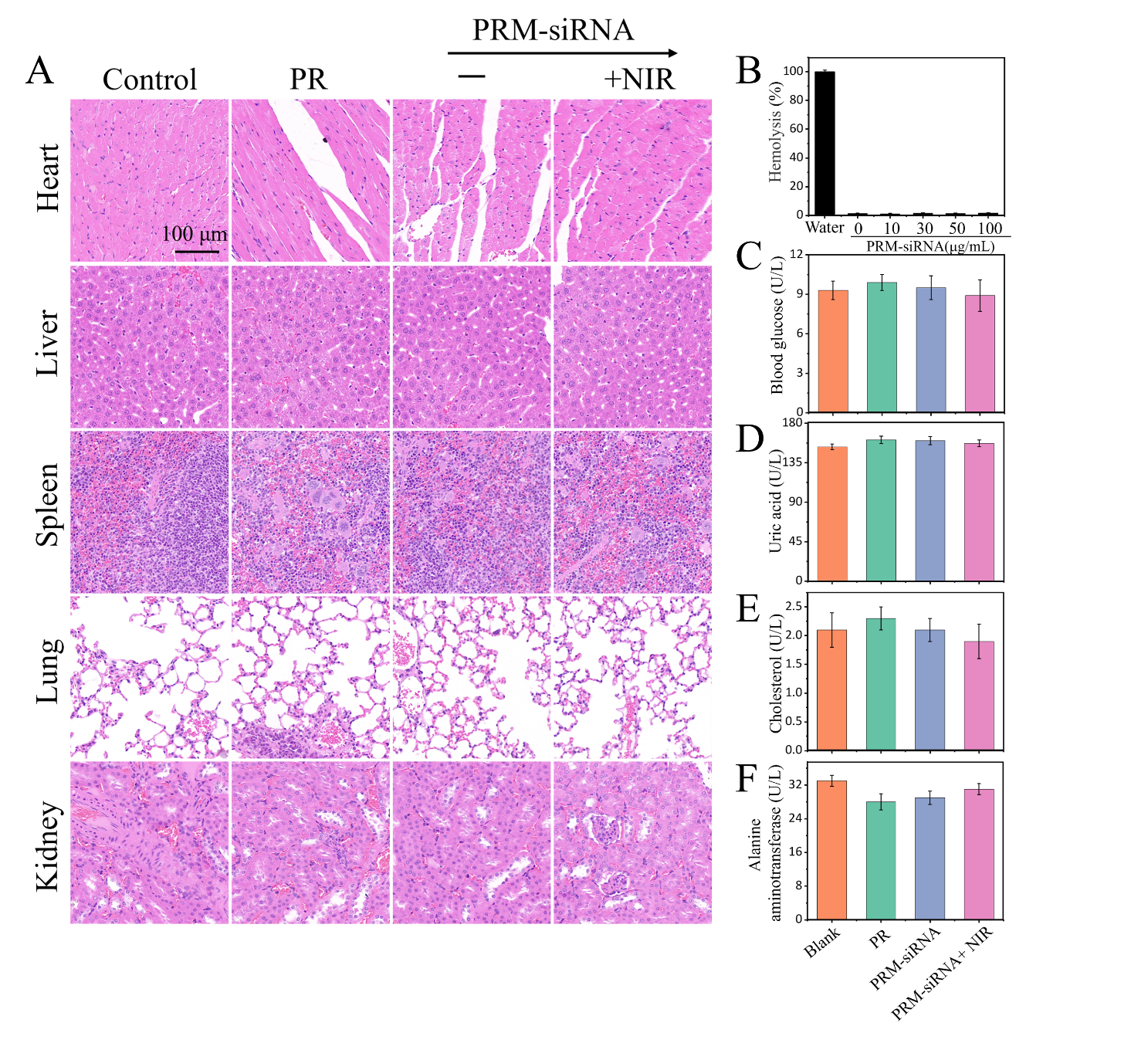


**Figure S9. Biosafety evaluation.** (A) H&E staining to observe histopathological changes in major organs. (B) Drug hemolysis rate, (C) blood glucose changes, (D) renal function indices, (E) cholesterol changes, and (F) liver function indices.


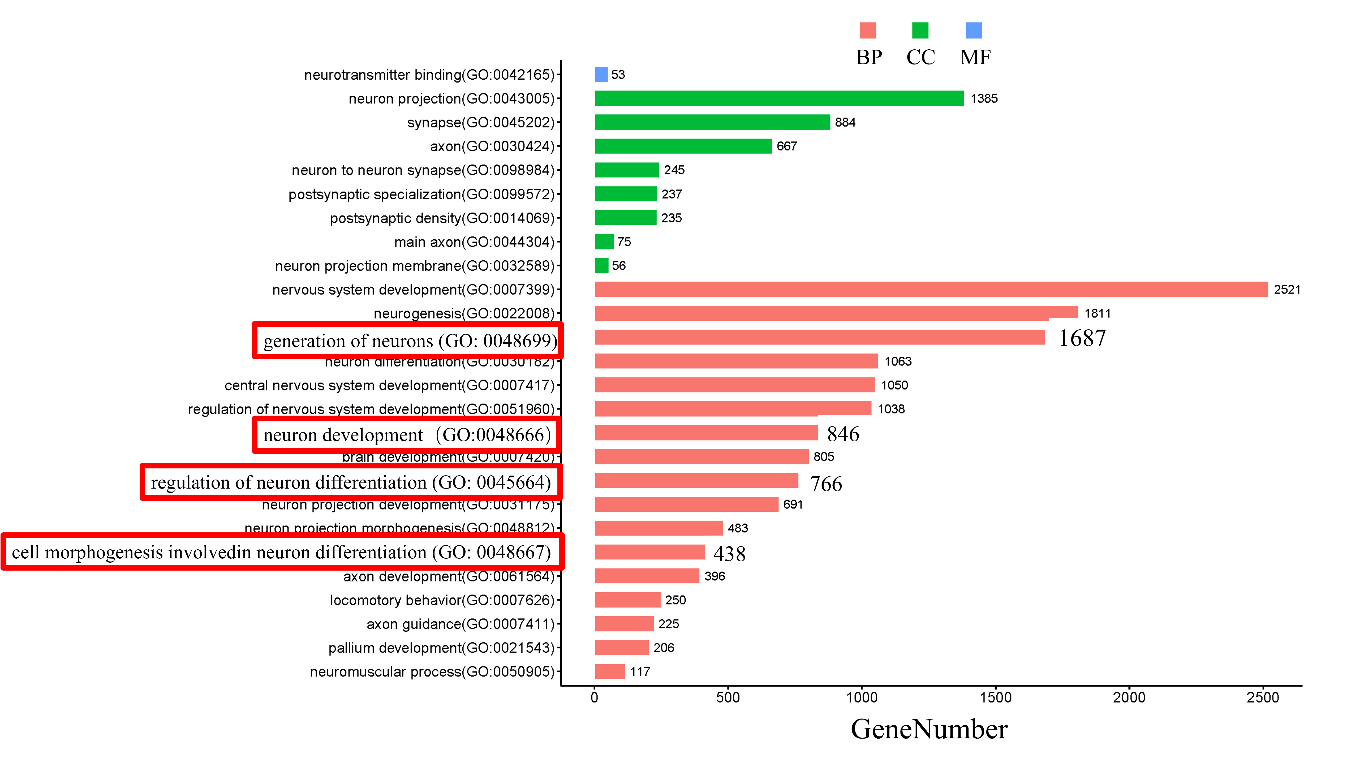


**Figure S10.** Most enriched GO terms (AD versus NPs). Genes were annotated in three main categories: biological process, cellular component, and molecular function.

**
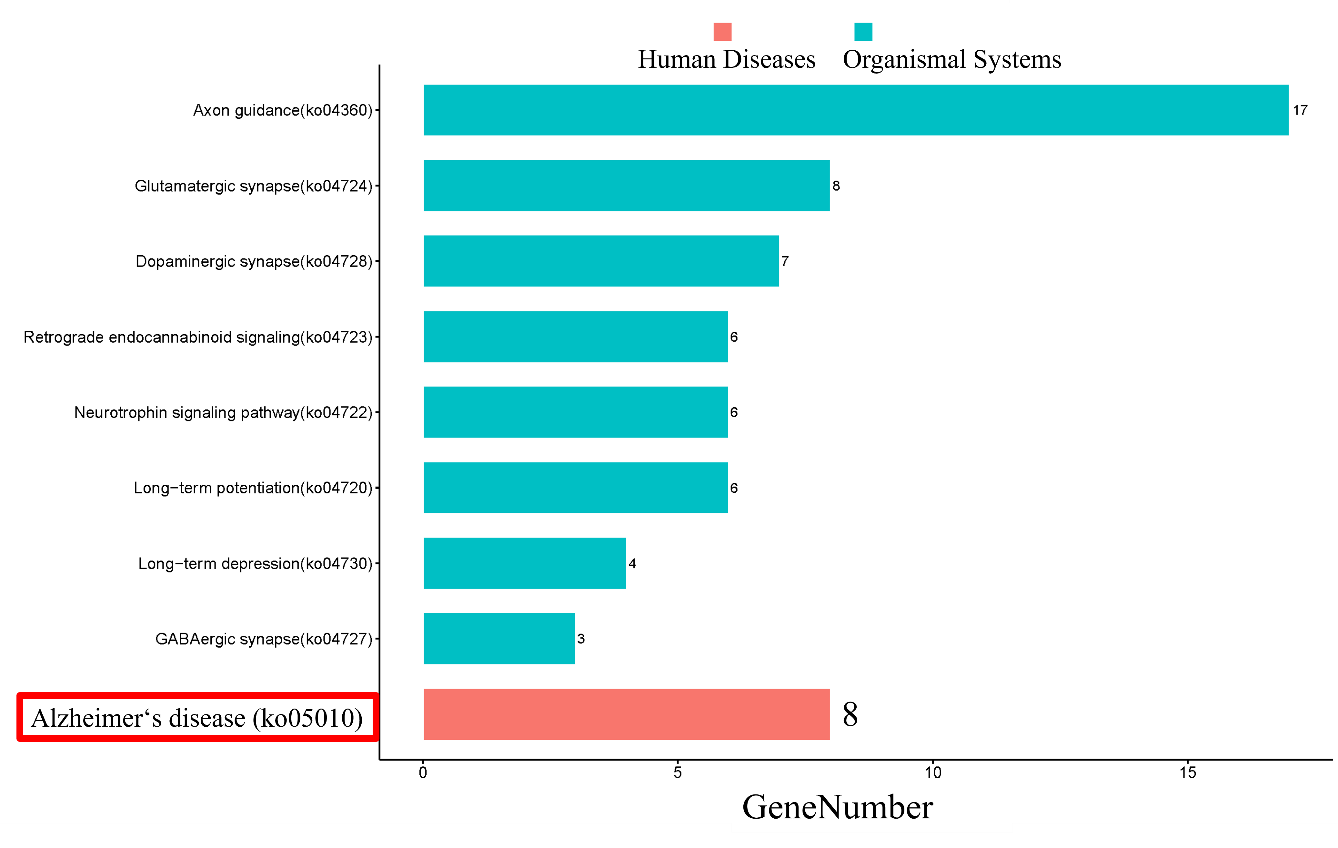
**

**Figure S11.** KEGG enrichment map (AD versus NPs). Red indicates human diseases, and blue indicates organismal systems.
